# Unraveling resistance mechanisms to the novel nucleoside analog RX-3117 in lung cancer: insights into DNA repair, cell cycle dysregulation and targeting PKMYT1 for improved therapy

**DOI:** 10.1186/s13046-025-03470-z

**Published:** 2025-07-24

**Authors:** Mahrou Vahabi, Geng Xu, Dzjemma Sarkisjan, Btissame El Hassouni, Giulia Mantini, Valentina Donati, Bing Wang, Giulia Lencioni, Richard J. Honeywell, Dongmei Deng, Sabrina Strano, Godefridus J. Peters, Giovanni Blandino, Elisa Giovannetti

**Affiliations:** 1 Department of Medical Oncology, Cancer Center Amsterdam, Amsterdam University Medical Center, Vrije Universiteit Amsterdam, Amsterdam, The Netherlands; 2 Unit of Pathological Anatomy 2, Azienda Ospedaliero-Universitaria Pisana, Pisa, Italy; 3 Cancer Pharmacology Lab, AIRC Start-Up Unit, Fondazione Pisana per la Scienza, Pisa, Italy; 4 Department of Preventive Dentistry, Academic Centre for Dentistry Amsterdam (ACTA), Amsterdam, The Netherlands; 5 Translational Oncology Research Unit, IRCCS Regina Elena National Cancer Institute, Rome, Italy; 6 Department of Biochemistry, Medical University of Gdansk, Gdansk, Poland

**Keywords:** Nucleoside analogs, Chemoresistance, Non-small cell lung cancer, PKMYT1, Cell cycle distribution, DNA repair

## Abstract

**Background:**

Nucleoside analogues are crucial in treating non-small cell lung cancer (NSCLC), but resistance hampers patient outcomes. The cytidine analogue RX-3117 shows promise in gemcitabine-resistant cancers, yet mechanisms underlying acquired resistance to this drug remain unexplored. This study includes a comprehensive investigation into RX-3117 resistance mechanisms by leveraging new preclinical models and cutting-edge genomic tools, including a CRISPR-Cas9 knockout screen and transcriptomics.

**Methods:**

NSCLC cell lines A549 and SW1573 were exposed to stepwise increasing concentrations of RX-3117 to establish stable resistant subclones, confirmed by SRB and clonogenic assays. Intracellular RX-3117 nucleotide levels were measured via LC/MS-MS, prompting the evaluation and modulation of the expression of key metabolic enzymes by Western blot and siRNA. A CRISPR-Cas9 screen identified genes whose loss increased RX-3117 sensitivity, while RNA-sequencing with differential expression analyses revealed resistance-related pathways, further investigated through cell cycle distribution, knock-out, and ELISA assays.

**Results:**

Resistant clones exhibited decreased accumulation of RX-3117 nucleotides, which however, was not associated to reduced expression of activation enzymes (UCK2, UMPK, CMPK, NME1/NDPK, RR1 and RR2). Instead, increased expression was observed in certain DNA repair and deactivation enzymes (NT5C3) but pharmacological inhibition and silencing of the latter did not circumvent resistance. Remarkably, a comprehensive approach with CRISPR-Cas9 screen highlighted DNA-repair and cell cycle determinants as key sensitizing genes. XL-PCR and RNA-sequencing confirmed aberrations in DNA-repair and pathways involved in cell cycle regulation. Knock-out and pharmacological inhibition validated the role of PKMYT1, a protein kinase involved in G2/M transition and genomic stability. RX-3117-resistant A549 cells showed enhanced sensitivity to the PKMYT1 inhibitor lunresertib and its synergism with RX-3117, suggesting further studies, especially in patients with high PKMYT1 expression who have significantly shorter survival rates, as observed in public databases and validated in an internal cohort of NSCLC patients.

**Conclusion:**

By integrating CRISPR-Cas9 with functional assays and transcriptomics, our study established a framework for decoding resistance mechanisms and highlights potential therapeutic strategies to enhance RX-3117 efficacy in NSCLC. We demonstrated for the first time that aberrant DNA repair and cell cycle dysregulation led resistance, identifying PKMYT1 as a promising target.

**Supplementary Information:**

The online version contains supplementary material available at 10.1186/s13046-025-03470-z.

## Background

The deoxycytidine analog gemcitabine has shown effectiveness in treating various tumor types, including lung and pancreatic cancer, in the past decades [1]. Nevertheless, a number of patients are inherently resistant or develop resistance during treatment [2] and are therefore in dire need of alternative treatment options.

Fluorocyclopentenylcytosine (RX-3117) is a novel oral antimetabolite currently under clinical evaluation for various cancer types. In a phase I study, the maximum tolerated dose and pharmacokinetics were determined in patients with advanced malignancies, paving the way for a subsequent phase II study (NCT02030067) [3, 4], showing its efficacy in advanced bladder cancer and relapsed or refractory pancreatic cancer patients. Additionally, a phase II trial demonstrated a progression-free survival (PFS) of 23.4 weeks (95% CI: 15.1–32.6) in 33 out of 38 patients treated with RX-3117 in combination with nab-paclitaxel as a first-line therapy for metastatic pancreatic cancer (NCT03189914) [5, 6].

Mechanistically, RX-3117 is transported into the cells by the human equilibrative nucleoside transporter-1 [7], and is activated to its mono-phosphorylated form by uridine-cytidine kinase 2 (UCK2) [8]. This activation is particularly advantageous, as UCK2 is selectively expressed in cancer and placental cells [9], suggesting a favorable toxicity profile. Additionally, unlike gemcitabine, RX-3117 is minimally deaminated by cytidine deaminase (CDA) [7], enabling effective oral administration [10]. RX-3117 monophosphate is further phosphorylated to di- and three-phosphate which are responsible for its cytotoxicity. As a cytidine analog RX-3117 inhibits RNA and DNA synthesis by incorporating into these macromolecules [7], although the exact metabolite that is incorporated into DNA has not been identified yet. Beyond this, RX-3117 has been shown to inhibit DNA methyltransferase 1 (DNMT1), leading to DNA hypomethylation and the reactivation of tumor suppressor genes [11]. These combined mechanisms, along with RX-3117’s distinct activation pathway and unique mechanism of action, likely explain its cytotoxic effects and its efficacy even in human gemcitabine-resistant cell lines and xenograft models [12]. However, as with all anticancer therapies, resistance remains a significant challenge, and elucidating the molecular mechanisms underlying RX-3117 resistance is crucial for its clinical development and for optimizing treatment strategies in resistant cancers.

Although the use of gemcitabine has declined in recent years with the advent of immunotherapy and targeted agents, it remains part of selected chemotherapy regimens for NSCLC, particularly in combination with platinum agents or in patients ineligible for other therapies [13], Thus, NSCLC is a key target for RX-3117 clinical development. To investigate the mechanisms of acquired resistance to RX-3117, we established multiple stable RX-3117-resistant cell clones. Using a multi-faceted approach, we examined cross-resistance to other cytidine analogs, and studied key enzymes involved in the metabolism of cytidine analogs, including those responsible for activation, deactivation, and DNA repair [2]. Additionally, we performed RNA sequencing of the resistant variants, complemented by a CRISPR-Cas9 drug screen [14], to identify genes whose loss increased sensitivity to RX-3117. These analyses revealed potential resistance-related pathways, which were further explored through cell cycle distribution analysis, knock-out experiments, and ELISA assays.

Interestingly, these studies pointed out the role of PKMYT1, a protein kinase involved in G2/M transition and genomic stability [15]. RX-3117-resistant A549 cells showed enhanced sensitivity to the PKMYT1 inhibitor lunresertib and its synergistic interaction with RX-3117, suggesting further studies, especially in patients with elevated PKMYT1 expression, which correlates with significantly reduced survival rates, as seen in public databases and corroborated by an internal cohort of NSCLC patients.

## Materials and methods

### Cell lines

The NSCLC cell lines A549 (adenocarcinoma), SW1573 (squamous cell carcinoma), and H460 (large cell carcinoma), and their corresponding resistant variants were cultured in Dulbecco’s Modified Eagle medium (DMEM), supplemented with 10% fetal bovine serum and 20 mM Hepes buffer, at 37 °C under 5% CO2. All cell lines were tested for Mycoplasma presence using the MycoAlert Mycoplasma detection kit (Lonza, Basel, Switzerland).

### Drugs and chemicals

RX-3117 and gemcitabine were dissolved in sterile water. Other compounds, including 5-azacytidine, and lunresertib, were dissolved in DMSO. Additional information about materials can be found in Supplementary Table 1.

### Evaluation of Inhibition of cell growth using the Sulforhodamine B (SRB) assay

Cellular sensitivity to RX-3117, lunresertib and drugs mentioned in the Supplementary Table 1, was measured with the SRB assay. Cells (3000–5000 cells per well) were seeded in 96-well plates and exposed to the compounds for 72 h, and inhibition of cell growth was determined as described previously [16]. Briefly, optical density (OD) was measured to evaluate cell growth inhibition, and IC50, the half-maximal inhibitory concentration, was determined through non-linear curve fitting or interpolation. Further details on the SRB assay, as well as the protocols about NT5C3 silencing and pharmacological inhibition are detailed in the Supplementary Methods. Finally, for drug combination analysis, the data were further evaluated using CalcuSyn, as described previously [17].

### Establishment of resistant cell lines

**Fig. 1:**
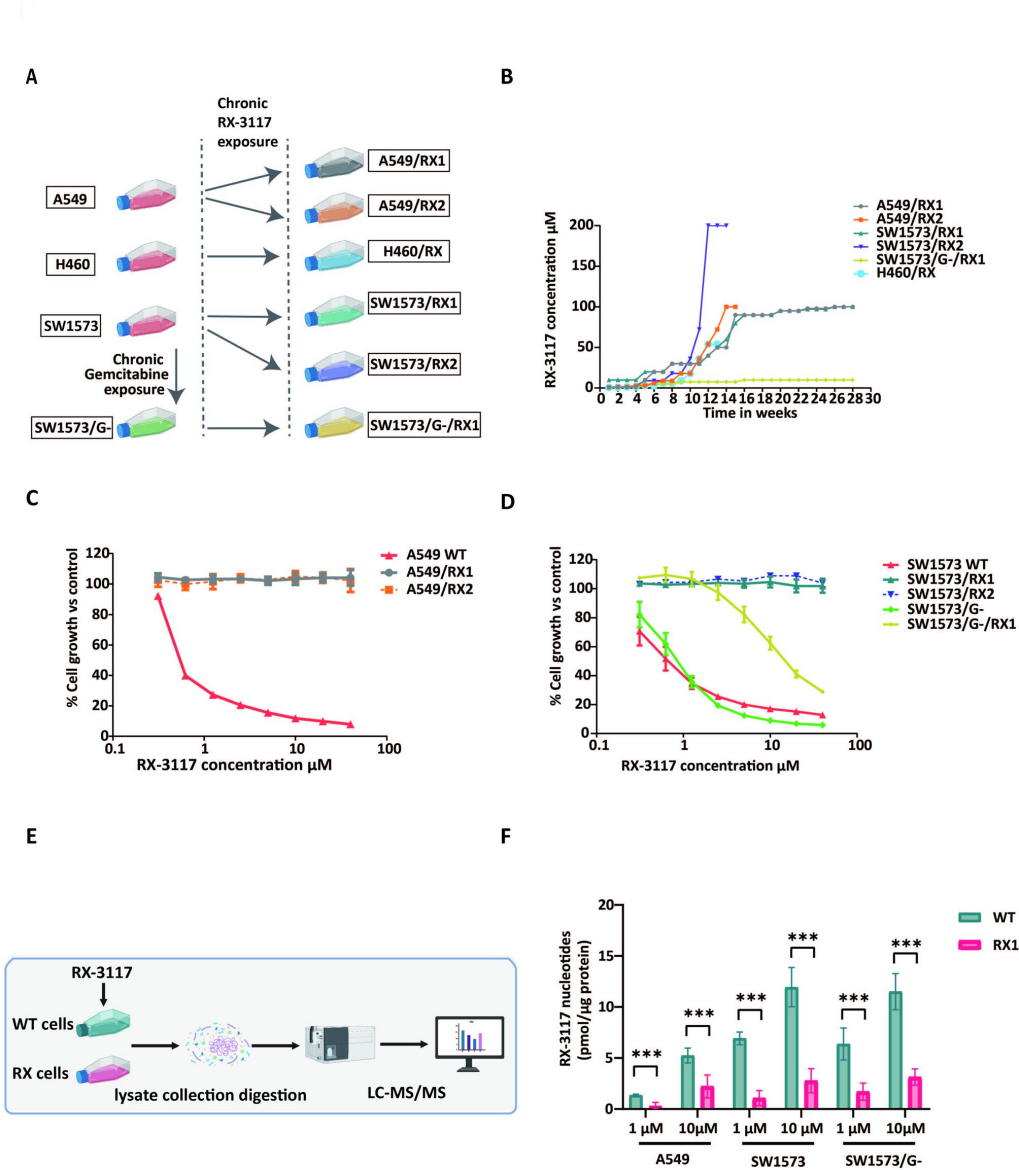
RX-3117 resistant NSCLC models and the role of nucleotide accumulation. (**A**) RX-3117 resistant cell lines were established by chronic exposure starting from A549 parental (wild type, WT), SW1573 WT, H460 WT cells and the gemcitabine resistant SW1573/G- cells. (**B**) Time course of exposure and stepwise increase in RX-3117 concentrations to produce resistant variants. (**C, D**) Growth inhibition curves of A549 (**C**) and SW1573 (**D**) resistant variants after 72-hr exposure to RX-3117. No growth inhibition was observed at the highest concentration of RX-3117 used, except for the SW/G/RX cell line. Results in the graph are from one representative experiment and bars represent the SD. IC50 values were calculated as mean of 3 independent experiments, each performed in triplicate. (**E**) Flow chart of the LC/MS-MS analysis. (**F**) Concentration dependent accumulation of RX-3117 nucleotides in A549 WT, SW1573 WT, SW1573/G- cells and depletion of RX-3117 nucleotides in RX-3117 resistant cells. Statistical significance was highlighted by asterisks, as follows: *P < 0.05, ***P < 0.001

The NSCLC cells A549, SW1573 and H460 were exposed to stepwise increasing RX-3117 concentrations, starting with the IC50. The concentration was increased (usually 2-fold) when the cells showed a normal duplication time which remained stable for 1–2 weeks. This process was continued until the maximum tolerated concentration was reached after 3–7 months. Parental cells never exposed to the drug were cultured in parallel with the resistant cells. Resistance induction was performed on two separate occasions, two years apart, resulting in two distinct variants named RX1 and RX2 [Fig. 1A]. To determine stable resistance RX1 and RX2 cells were grown in drug-free medium and baseline growth and resistance to the maximum tolerated concentration was analyzed by SRB at regular intervals for up to two months.

The resistance factor (RF) was regularly determined using the SRB assay, calculated by dividing the IC50 value of the resistant cells by that of the corresponding wild-type cells. If the resistant cells did not achieve 50% growth inhibition, the RF was determined by dividing the highest concentration tested in the corresponding wild-type cells.

Additionally, the gemcitabine-resistant variant SW1573/G-, harbouring a deoxycytidine kinase deficiency [18], was further made resistant to RX-3117, resulting in the double-resistant SW1573/G-/RX variant [Fig. 1A].

### Measurement of nucleotide accumulation by LC-MS/MS

Nucleotide accumulation was measured by liquid chromatography-tandem mass spectrometry (LC-MS/MS) following treatment of cells with 1 or 10 µM RX-3117 for 24 h. Cells were processed to isolate both free cytosolic RX-3117 and phosphorylated RX-3117, which was degraded to its free form for total RX-3117 quantification. Analyses were performed using an API5500 Triple Quadrupole mass analyzer equipped with a Prodigy 5 ODS-2 column (Phenomenex). Total phosphorylated RX-3117 levels were calculated by subtracting free cytosolic RX-3117 from the total RX-3117 content. The methods were based on previously validated protocols for nucleotide analysis [19, 20] and are detailed in the Supplementary Methods.

### Analysis of protein expression by Western blot

Briefly, cells were harvested and lysed in a diluted cell lysis buffer supplemented with 1 mM phenylmethylsulfonyl fluoride, a chemical compound commonly used in biochemical research as a serine protease inhibitor [8]. Protein concentrations were determined using a colorimetric assay (Bio-Rad). Protein lysates were separated on Bio-Rad Mini-Protean TGX™ precast gels and transferred onto PVDF membranes. Additional information and details about the antibodies are provided in Supplementary Table 1. Detection of signals was performed using an Odyssey Infrared Imager (Li-COR Biosciences) with InfraRedDye secondary antibodies or HRP-linked antibodies. Band quantification was conducted using ImageJ software, as described previously [11].

### Extra-long PCR (XL-PCR)

**Fig. 2:**
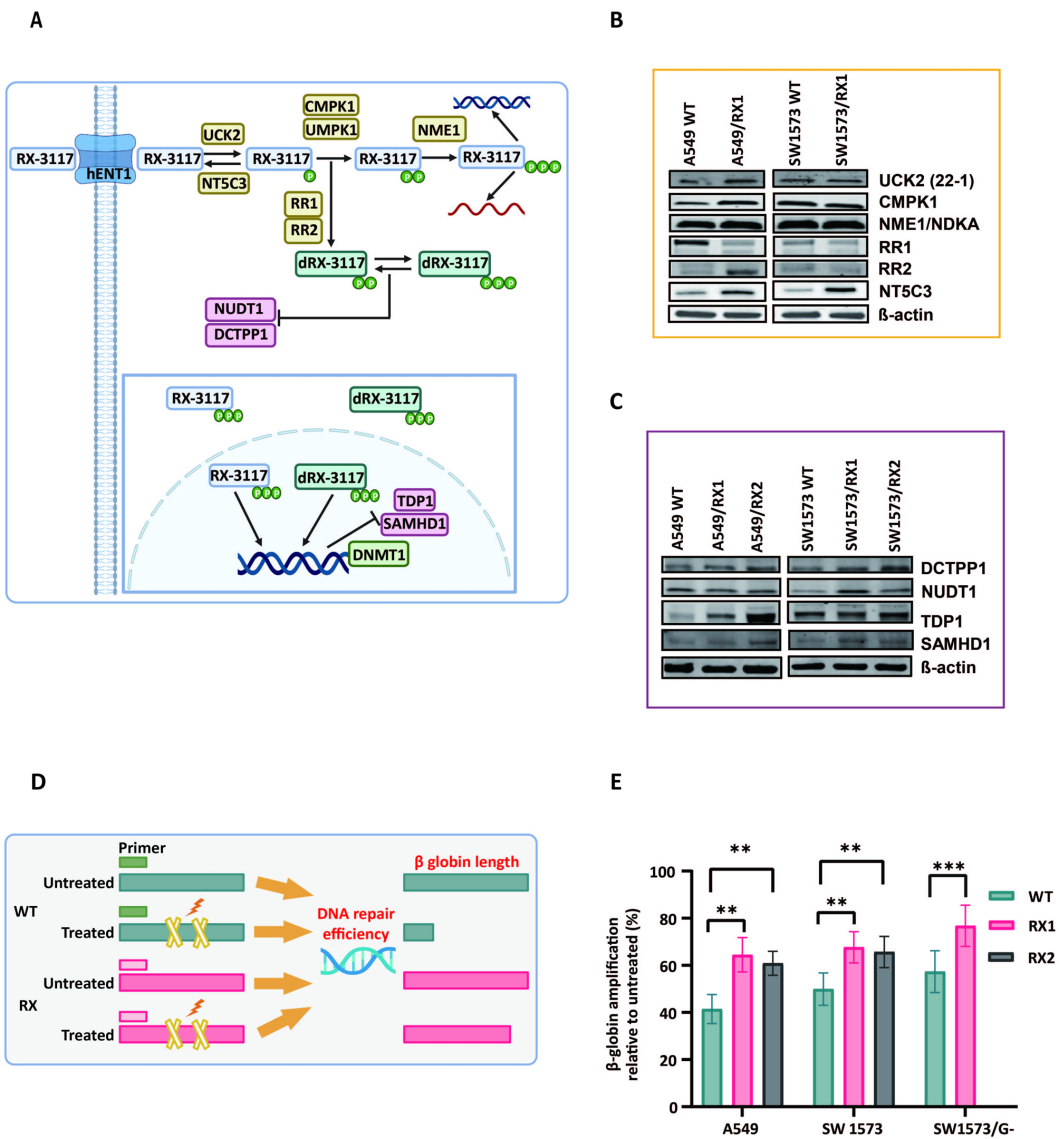
Role of metabolism and DNA repair enzymes in RX-3117 acquired resistant cells and extent of accumulated DNA damage by XL-PCR. (**A**) Putative mechanism of action of RX-3117 and assumed components of the RX-3117 pathway that could contribute to resistance. (**B**) No common modulation of protein expression (i.e. increased or decreased expression) was observed for the resistant variants when considering the activation enzymes, UCK 2 clone 22 − 1 (UCK2 (22 − 1)), cytidine/uridine monophosphate kinase 1 (CMPK1), NME/NM23 nucleoside diphosphate kinase 1 (NME1/NDKA). A decreased expression of ribonucleotide reductase 1 and increased expression of ribonucleotide reductase 2 was observed in A549 cells (RR1 and RR2). Conversely, significantly increased expression of NT5C3 in both resistant variants was observed (representative blots, with 30 µg protein loading per sample, using β-actin as a loading control). (**C**) Increased expression of DCTPP1, NUDT1, TDP1, SAMHD1 was detected in both resistant variants, though more pronounced in RX1 cells (representative blots, with 40 µg protein loading per sample using β-actin as loading control). (**D**) Flow chart of the methodology used for the XL-PCR. (E) β-globin amplification as a measure of accumulated DNA damage was determined in A549 WT, SW1573 WT, SW1573/G- cells and RX-3117 resistant cells. Data represent the means ± SD from 3 individual experiments. Statistical significance was highlighted by asterisks, as follows: *P < 0.05, **P < 0.01, ***P < 0.001

To induce DNA damage, cells were exposed to UV irradiation for 30 min by placing them directly under the bulbs of a UV Stratalinker 1800, which emits light at a wavelength of 254 nm (Stratagene). DNA was then extracted from adherent cells using TriZol Reagent, and XL-PCR was performed to evaluate accumulated DNA damage within an extended region targeted by specific primers, as previously described [21] and illustrated in Fig. 2.

### Clustered regularly interspaced short palindromic repeats (CRISPR) Cas9 screen

**Fig. 3:**
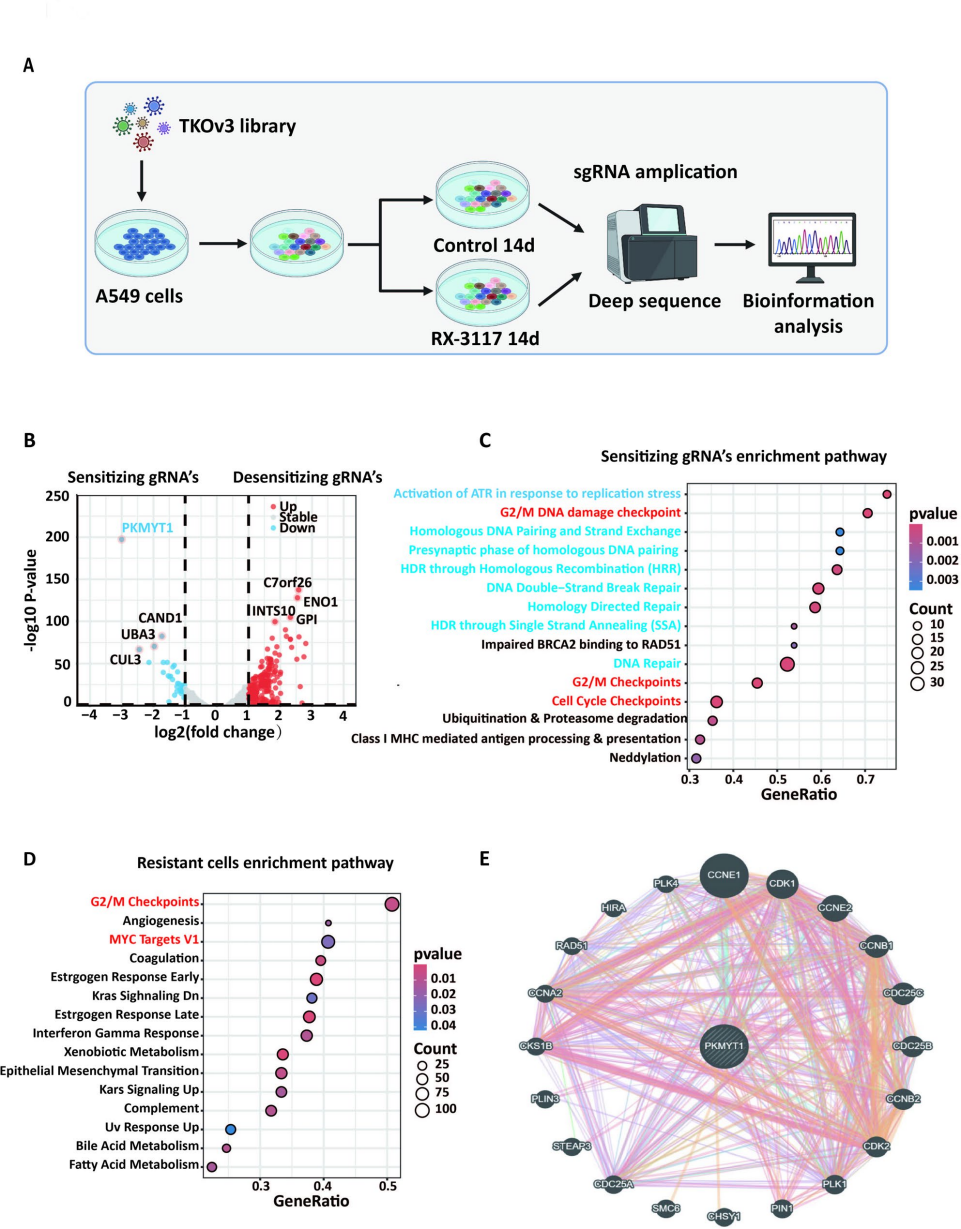
CRISPR library screening and transcriptomics identified PKMYT1 and associated cell cycle and DNA repair genes pathways as drivers for RX-3117 resistance. (**A**) Graphical representation of the workflow for the CRISPR screen performed in A549 cells with TKO v3 whole-genome gRNA library. (**B**) Volcano plot showed the significant sensitizing and desensitizing genes as reflected by the indicated sensitizing and desensitizing gRNAs. The fold change (Log2) is plotted on the x-axis and the significance (− Log10 p-value) is plotted on the y-axis. The most significant sensitizing gene (PKMYT1) was highlighted. (**C**) Top 15 pathways enriched in sensitizing genes, as determined by the GSEA analysis (*P < 0.05). Red color indicates cell cycle pathway, and blue color indicates DNA repair pathway. (**D**) Top 15 pathways were enriched in the RNA-seq data between A549-WT and A549-RX1 cells, using the GSEA analysis, with red color indicating cell cycle pathway. (**E**) The GeneMANIA network showed that PKMYT1 is closely associated with key genes regulating the cell cycle

The CRISPR-Cas9 drug screen was performed as described previously [14, 22]. A549 cells were infected with the pooled ToroCnto KnockOut v3 (TKOv3) lentiviral library at a multiplicity of infection (MOI) of 0.3 [23]. Subsequently, puromycin was added to select for positively transduced cells. The A549 cells were then treated with RX-3117 at an initial concentration of IC50, which was gradually increased as shown in Fig. 3. After 14 days of RX-3117 treatment, the cells were harvested. The sgRNA sequences from both the treated and untreated populations were sequenced and analyzed using DrugZ [24].

### RNA sequencing and analysis

Total RNA was extracted from cells using the MiRVAna kit. Library preparation was performed using the Illumina TruSeq Stranded total RNA Library Prep gold Kit (Illumina Inc., San Diego, USA) and Agencount AMPure XP beads (Beckman Coulter, Brea, USA). Analyses of library concentration, RNA sequencing and bioinformatics analysis pipeline were performed as described previously [16, 25]. Sequences were mapped to the human genome (GRCh38) using the STAR alignment tool (version 2.5.3a).

Differential gene expression analysis was performed using edgeR, and visualization was done using ggplot, both available in the Bioconductor repository for R software. The gene set enrichment (GSEA) was performed. The interaction network was built and visualized in GeneMANIA [26].

### RNA extraction and reverse-transcription quantitative PCR (RT-qPCR)

RNA isolation was performed using the TRIzol protocol. A total of 0.5 µg of RNA was reverse transcribed into complementary DNA (cDNA) using the First Strand cDNA Synthesis Kit (Thermo Scientific), following the manufacturer’s instructions. The resulting cDNA samples were diluted 10-fold in nuclease-free water. RT-qPCR was carried out using the SsoAdvanced Universal SYBR Green Supermix with a CFX96 Real-Time System (Bio-Rad). Details of the primers sequences are provided in Supplementary Table 1.

### Analysis of modulation of protein expression by enzyme-linked immunosorbent assays (ELISA)

Detection of PKMYT1 and CDK1 in cell lysates was performed using the Total PKMYT1 ELISA Kit (ab279891, Abcam, Cambridge, UK) and the Human Phospho-CDK1 (Y15) and Total CDK1 ELISA Kits (RayBiotech, Peachtree Corners, USA), following the manufacturers’ protocols. Briefly, cell lysates were prepared and added to the appropriate ELISA plates, and after the incubation and washing steps as per the protocols, the absorbance was measured at 450 nm using the BioTek Synergy HT plate reader (BioTek Instruments Inc.).

To calculate the concentrations of PKMYT1, phospho- and total-CDK1, the blank OD values were subtracted from the OD values of the samples to account for background noise. A standard curve was generated for each analyte, and sample concentrations were interpolated from the respective curves, according to manufacturer’s protocol and previous studies [27].

### Creation of PKMYT1 knockout cells and colony formation assay

To generate PKMYT1 knockout A549 cells using the CRISPR-Cas9 system, LentiCRISPR-V2 plasmids containing guide RNAs (gRNAs) targeting the PKMYT1 gene were constructed. The gRNA sequences were designed to target the PKMYT1 gene. The LentiCRISPR-V2 plasmids were then transfected into A549 cells using Lipofectamine 3000 (Thermo Fisher Scientific). Forty-eight hours post-transfection, cells were selected with puromycin. Single clones were isolated by limiting dilution and expanded for further analysis. Knockout efficiency was confirmed by quantitative PCR and ELISA assay.

A549 wildtype, knockout cells (clone KO1, KO2) and resistant variants (RX1, RX2) were seeded in 6-well plates at a low density of 1000–3000 cells per well to ensure sufficient space for colony formation. After allowing the cells to adhere for 24 h, they were treated with different concentrations of RX-3117 (0.5 µM or 1 µM) and lunresertib (280 nM), either individually or in combination, depending on the experimental conditions. The medium containing the drugs was replenished every 3–4 days to support cellular growth and maintain a consistent drug exposure. Treatment continued for 10–14 days, depending on the growth rate of the cells, until colonies became visible to the naked eye.

Colony formation was assessed to evaluate clonogenic survival following treatment. After treatment, cells were fixed with methanol, stained with crystal violet, and colonies consisting of at least 50 cells were counted manually or via image analysis software. Results were expressed as a percentage of colony formation relative to untreated controls. Further details on this method are provided in the Supplementary Methods. Each experiment was performed in triplicate to ensure reproducibility and statistical robustness.

### Cell cycle distribution

Cell cycle distribution was analyzed after 24-hour treatment with 1 µM RX-3117 for A549, SW1573 and 10 µM for the resistant variants A549/RX1 and SW1573/RX1. Cells were fixed, stained with propidium iodide, and analyzed using a flow cytometer to assess changes in cell cycle phases as reported previously [28]. Detailed methodology is provided in the Supplementary Methods. Each experiment was conducted in triplicate for reproducibility.

### Analysis of PKMYT1 by immunohistochemistry (IHC)

PKMYT1 expression was analyzed by immunohistochemistry (IHC) in tissue microarrays (TMAs) constructed from formalin-fixed, paraffin-embedded specimens of patients who underwent radical surgical resection for primary NSCLC. The TMAs were created using a TMA instrument (Beecher Instruments, Micro-Array Technologies, Silver Spring, MD). After de-paraffinization, rehydration, and antigen retrieval using a pressure cooker with EDTA, the sections were incubated overnight with a rabbit monoclonal antibody specific for PKMYT1 (Cell Signaling Technology #4282). The sections were then treated with a horseradish peroxidase-conjugated secondary antibody and developed with 3,3’-diaminobenzidine (DAB), visualizing the antibody-antigen binding with a brown precipitate. The total IHC score was obtained by summing the staining intensity and positive range scores, providing a comprehensive measure of PKMYT1 expression levels by staining intensity strength and distribution [29]. Full details of the procedure, including scoring and analysis methods, are provided in the Supplementary Methods.

### TCGA and patient survival analysis

To assess PKMYT1 expression in lung cancer, gene expression data for the TCGA lung cancer cohort (TCGA-LUAD and TCGA-LUSC) were downloaded from the TCGA database (http://gdc.cancer.gov/). The raw counts were normalized using DESeq2. A Student’s t-test was then employed to identify statistically significant differences in gene expression between normal and tumor tissues. Analysis of patient survival were performed both in the TCGA database and in an internal clinical cohort, including patients from previous multicenter studies [30]. Overall survival and relapse-free survival were calculated from the date of pathologic diagnosis (i.e., the date of surgery/biopsy) to the date of death or relapse. The Kaplan–Meier method was used to plot survival curves and statistical differences were evaluated by univariate Cox proportional hazards regression analysis using the survival R package. Information on ethics approval is provided in the paragraph on ethics approval and consent to participate.

### Statistics

All experiments were performed in triplicate and repeated at least twice and data are expressed as mean ± SEM, unless otherwise specified. The statistical analyses were performed with GraphPad Prism version 9 (Intuitive Software for Science). Comparisons between two groups were performed with a two-tailed unpaired Student’s t-test. For multiple groups comparisons an ordinary one-way ANOVA multiple comparison test with Dunnet’s post-hoc test was used, unless otherwise specified in figure legends. Statistical significance was set at *p* < 0.05.

## Results

### Establishment of RX-3117 resistant NSCLC cells and evaluation of their resistance to other cytidine analogs

We utilized multiple NSCLC cell lines to establish RX-3117-resistant models [Fig. 1A]. Most experiments were conducted in A549 (adenocarcinoma) and SW1573 (squamous) cells, representing the main histological subtypes of NSCLC typically treated with standard immuno-/chemotherapy regimens, including cisplatin and gemcitabine.

At baseline, both cell lines were sensitive to RX-3117, with IC50 values of 0.5 ± 0.008 µM for A549 and 0.6 ± 0.15 µM for SW1573 [Table 1]. Resistance was induced by culturing the cells in stepwise (2-fold) increasing concentrations of RX-3117, starting at their respective IC50 values. Cells were cultured at each concentration until their growth rate matched that of untreated cells, after which the concentration was doubled again [Fig. 1B]. This process was repeated until stable resistance was achieved, which required 12 to 28 weeks depending on the cell line.

**Table 1:** Establishment of resistant variants of the NSCLC cells. IC_50_ and respective RF values to RX-3117

Cell line		IC_50_ (µM) ± SEM	RF values
A549		0.5 ± 0.008	NA
A549/RX1		> 400	800
A549/RX2		300 ± 19.7	600
SW1573		0.6 ± 0.1	NA
SW1573/RX1		> 400	667
SW1573/RX2		292 ± 10.2	487
SW1573/G-		1.0 ± 0.1	NA
SW1573/G-/RX		9.1 ± 1.1	9

Abbreviations: IC_50_, Half maximal inhibitory concentration; RF, Resistant factor; SEM, standard error of the mean

From the A549 and SW1573 parental lines, two independent RX-3117-resistant variants were established: A549/RX1 (RF: 800), A549/RX2 (RF: 600), SW1573/RX1 (RF: 667), and SW1573/RX2 (RF: 487), with an interval of two years between their establishment [Fig. 1C and D]. For both cell lines, the RX1 variants exhibited greater resistance than the RX2 variants, as indicated by their higher RF values.

A similar time was used to create a resistant variant of the SW1573/G- cells, resulting in the double-resistant SW1573/G-/RX variant with IC50 values of 1.0 ± 0.1 and 9.1 ± 1.1 µM, respectively. Of note, these cells exhibited lower resistance (RF: 10), more likely because resistance to RX-3117 developed in a background already adapted to gemcitabine, limiting the evolutionary space for additional resistance mechanisms, as reported for other anticancer drugs [31].

From the large cell lung cancer cell line H460 (baseline IC_50_, 0.05 ± 0.01 µM) we established an additional resistant variant H460/RX (RF > 800). Interestingly this variant lost its resistance after approximately two lost months of culture in drug-free medium. All reported data on this cell line were obtained during the resistant stage.

The activity of other nucleoside analogs used in the clinical setting, such as gemcitabine and azacytidine, was tested in the resistant variants. These analyses showed a trend towards a reduced sensitivity in most models, as reported in Supplementary Table 2.

### Accumulation of RX-3117 nucleotides

Since we previously demonstrated a relationship between RX-3117 nucleotide formation and sensitivity to RX-3117 in various unselected cell lines [7], we hypothesized that a decreased accumulation of RX-3117 nucleotides could serve as a resistant mechanism. Of note, this is a common resistance mechanism for many antimetabolites [32]. Using LC/MS-MS, we assessed the accumulation of RX-3117 and its nucleotides after 24 h exposure to 1 or 10 µM RX-3117 in the A549, SW1573 [Fig. 1E and F] and their resistant RX1 variants. In the wild type cells, RX-3117 exposure resulted in a concentration-dependent accumulation of RX-3117 nucleotides. However, all resistant cells derived from both A549 and SW1573 cells exhibited a significant reduction in accumulation of RX-3117 nucleotides [Fig. 1F]. Similar results were observed in the SW1573/G-/RX variant [Fig. 1F].

As a decrease in nucleotide accumulation of antimetabolites is often associated with reduced uptake, impaired activation, or increased degradation [33], we focused our following experiments on the key enzymatic determinants of RX-3117 metabolism.

### Analyses of metabolism modulation: expression of activation and deactivation enzymes

For RX-3117, depletion of its (deoxy)nucleotides could potentially be caused by either a decreased uptake, impaired activation (by UCK2 or monophosphate or diphosphate kinases), or increased degradation [Fig. 2A]. To investigate these possibilities, we analyzed the protein expression of several of key enzymes involved in RX-3117 metabolism, as well as that of ribonucleotide reductase (RR), which could mediate the conversion of RX-3117-diphosphate to its deoxy-diphosphate form, and deactivation enzymes such as 5’-nucleotidase (NT5C3). We first examined whether a deficiency of UCK2, the main activation enzyme for RX-3117 [8],, might account for the observed depletion of RX-3117 nucleotides. As shown in the representative blots in Fig. 2B and quantified in Fig. S1, no difference in the protein expression level was observed between wild type and resistant models.

Next, we investigated the expression of putative nucleotide kinases, that might be involved in further phosphorylation of RX-3117-monophosphate [34, 35]. No changes were observed in the expression levels of NME1/NDKA (NME/NM23 nucleoside diphosphate kinase 1). However, a slight increase in CMPK1 expression was detected in the A549 RX1-resistant model. Furthermore, ribonucleotide reductase 1 (RR1) showed a decrease in the A549/RX1 cells, while RR2 expression remained unchanged in the SW1573-resistant variant but increased in A549/RX1 [Fig. 2B and S1]. Taken together, these findings suggest that none of the investigated enzymes are likely responsible for the depletion of RX-3117 nucleotides and can be excluded as potential contributors to RX-3117-induced resistance.

We also examined the expression levels of the inactivation enzyme NT5C3. At the protein level, NT5C3 expression was increased in both resistant cell lines compared to their parental counterparts [Fig. 2B]. Due to the elevated NT5C3 expression, we further investigated its role using the NT5C3 inhibitor diethylpyrocarbonate (DEPC), and siRNA targeting NT5C3 [36]. Significant changes in RX-3117 nucleotide levels were observed after NT5C3 silencing, resulting in 75% inhibition of mRNA expression [Fig. S1B, S1C]. However, the effects of both DEPC and siRNA on chemosensitivity were minimal in both resistant models [Fig.S1D].

### Analysis of expression levels of potential activity of DNA repair enzymes

Since decreased expression of metabolic enzymes did not account for the depletion of RX-3117 nucleotides, we investigated the expression of potential activity and DNA repair enzymes [Fig. 2C and Fig. S3]. Cytidine analogs are known substrates for degradation and DNA repair enzymes, including DCTPP1 (deoxycytidine-triphosphatase 1) [37], NUDT1 (Nudix hydrolase, also known as MTH1) [38], and TDP1 (tyrosyl-DNA phosphodiesterase 1) [39].

At the protein level, both DCTPP1 and NUDT1 exhibited a trend toward increased expression compared to parental cells; however, these changes did not reach statistical significance. Similarly, the DNA repair enzyme TDP1 appeared upregulated in the RX-3117-resistant variants, with more noticeable increases in A549/RX1 and SW1573/RX1, correlating with their higher resistance factors (RF). Given the role of SAMHD1 in nucleoside triphosphate metabolism [38], we also assessed its expression, which showed a modest, non-significant increase specifically in A549/RX1 cells [Fig. 2C and Fig. S1E]. Thus, the densitometric analysis suggests that, although the magnitude of upregulation varies across cell lines and clones, certain DNA repair genes show a non-significant trend toward increased expression in the resistant variants.

Then, to quantitatively evaluate the extent of accumulated DNA damage, we employed XL-PCR using a long-range PCR fragment, a technique commonly used to assess the effects of DNA-damaging agents, such as UV light. Accumulation of DNA damage results in a reduction in β-globin amplification [40] [Fig. 2D]. As shown in Fig. 2E, the upregulation of DNA repair enzymes in the RX-3117-resistant variants was associated with higher β-globin expression levels in the resistant variants compared to the wild-type A549, SW1573, and SW1573/G- cells.

### CRISPR library screening and expression analysis identified PKMYT1 as a critical gene involved in RX-3117 sensitivity

In order to identify the critical genes associated to RX-3117, we performed a full genome CRISPR-Cas9 knockout library screening in NSCLC. As shown in Fig. 3A, the human TKO v3 library was successfully cloned into A549 cells to create a pool of mutant cells. It is hypothesized that knocking out RX-3117 resistance-suppressing genes (desensitizing genes) will enhance the development of resistance, while knocking out RX-3117 resistance-driving genes (sensitizing genes) will make NSCLC cells sensitive to RX-3117-induced cell death. Compared to the untreated group, 194 genes were identified as RX-3117 sensitizing genes and 815 genes were identified as desensitizing genes in A549 cells after 14 days of RX-3117 treatment. The volcano plot [Fig. 3B] depicted the four most RX-3117 sensitizing genes: PKMYT1, CAND1, UBA3, and CUL3, and the four most RX-3117 desensitizing genes: C7orf26, ENO1, INT510, and GP1. Among these, protein kinase membrane-associated tyrosine/threonine 1 (PKMYT1, also known as Myt1) emerged as a particularly interesting gene, as it was the most significant sensitizing factor for RX-3117. PKMYT1 plays a key role in cell cycle regulation and DNA repair, as it is a member of the WEE1 protein kinase family [15, 41]. GSEA analysis showed that the sensitizing genes were predominantly enriched in cell cycle pathways and DNA repair pathways [Fig. 3C], while desensitizing genes were predominantly distributed in ribosome translation related pathways [Fig. S2].

Next, we performed RNA sequencing analysis to identify differentially expressed genes between wild-type and RX-3117-resistant NSCLC cell lines. As shown in Fig. 3D, compared with A549 parental (wild type, A549-WT) cells, the most significantly altered pathways in A549/RX1 cells were the G2/M checkpoint pathway and MYC targets v1 pathway. In addition, we also found that the cell cycle related pathways were enriched in the SW1573/RX1 and H460/RX cells (Fig. S4A, S4B). GeneMANIA analysis revealed that PKMYT1 potentially interacts with 20 proteins, including CCNE1, CDK1, CCNE2, UBE2N, RNF126, CDC25C, CDC25B, CCNB2, CDK2, and PLK1, most of which are involved in cell cycle regulation [Fig. 3E]. In summary, PKMYT1 may be a key gene influencing RX-3117 sensitivity and resistance, with RX-3117 resistance strongly associated with cell cycle and DNA repair pathways.

### Functional validation of the role of the cell cycle regulator PKMYT1 in RX-3117 sensitivity

**Fig. 4:**
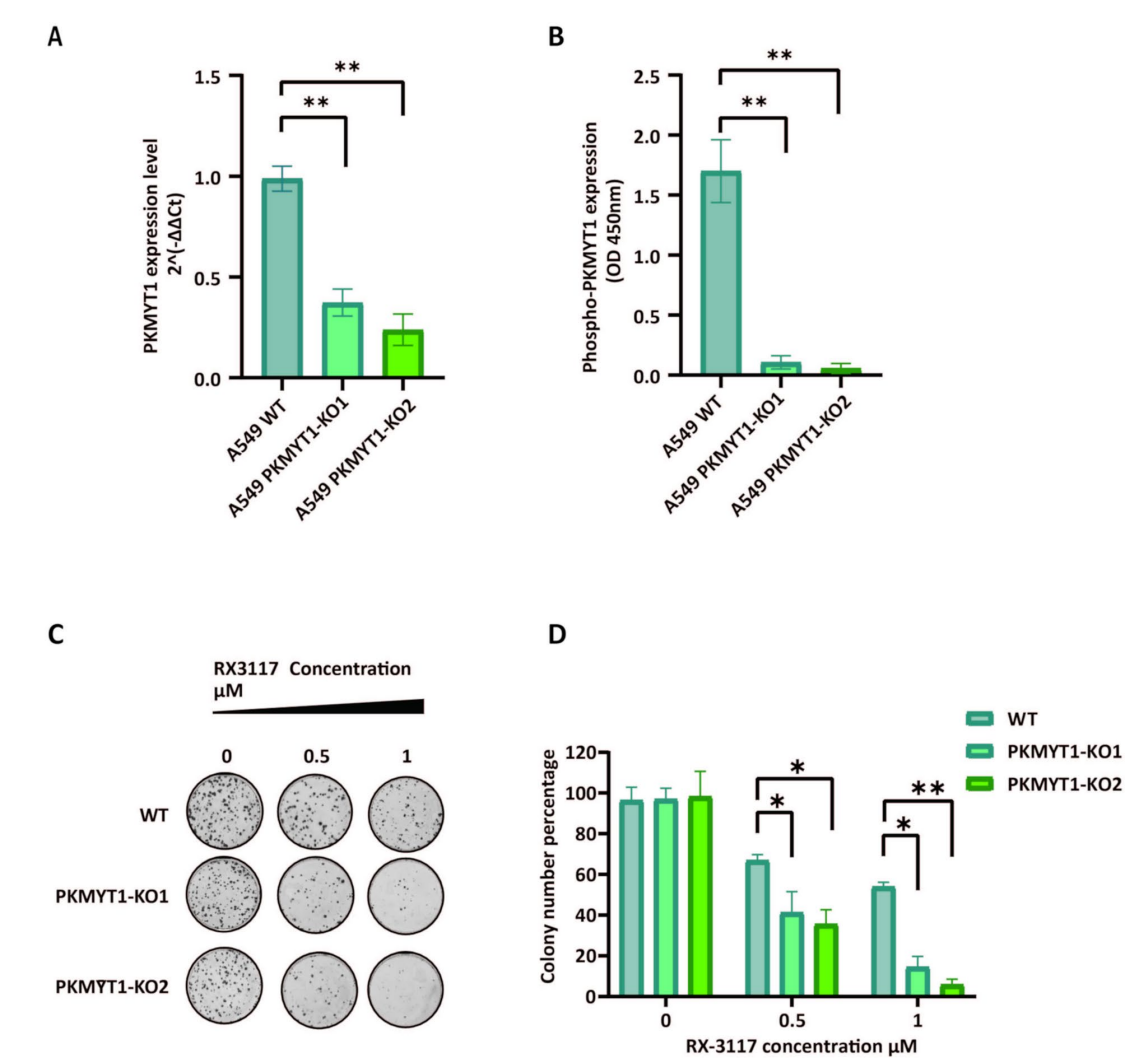
Validation of CRISPR Cas9 knockout status and the effect of cell cycle regulator PKMYT1 on RX-3117 sensitivity (**A**) Expression level of PKMYT1 by q-RT-PCR in A549 WT and single knock out clones KO1 and KO2. (**B**) Enzyme-Linked Immunosorbent Assay (ELISA) of phospho-PKMYT1 in A549 WT and KO1 and KO2 clones. (**C, D**) Colony formation assay performed on A549 WT and KO1 and KO2 clones, treated with different concentrations of RX-3117 in comparison with the negative control. Histogram bars show the means and SD of at least three experiments. Statistical significance was highlighted by asterisks, as follows: *P < 0.05; **P < 0.01

As previously shown, the top sensitizing gRNA hit was the protein kinase PKMYT1 [Fig. 3B]. To investigate its role in RX-3117 resistance, we first constructed an A549 PKMYT1 knockout cell line using CRISPR-Cas9. The knockout clones were then validated by assessing PKMYT1 expression through quantitative PCR and ELISA. Quantitative PCR results showed a significant decrease in PKMYT1 expression in the two knockout clones, KO1 and KO2 [Fig. 4A]. Additionally, ELISA results confirmed a reduction in total PKMYT1 protein levels in both clones, as indicated by the lower absorbance at 450 nm [Fig. 4B]. To further confirm the CRISPR-Cas9 knockout and the role of PKMYT1 in RX-3117 sensitivity, we performed a colony formation assay [Fig. 4C and D]. As shown in Fig. 4C, knockdown of PKMYT1 increased sensitivity to RX-3117, leading to a decrease in colony formation at 0.5 and 1 µM concentrations of RX-3117 compared to untreated cells.

### Effect of RX-3117 on cell cycle distribution in wild type and RX-3117 resistant cells

**Fig. 5:**
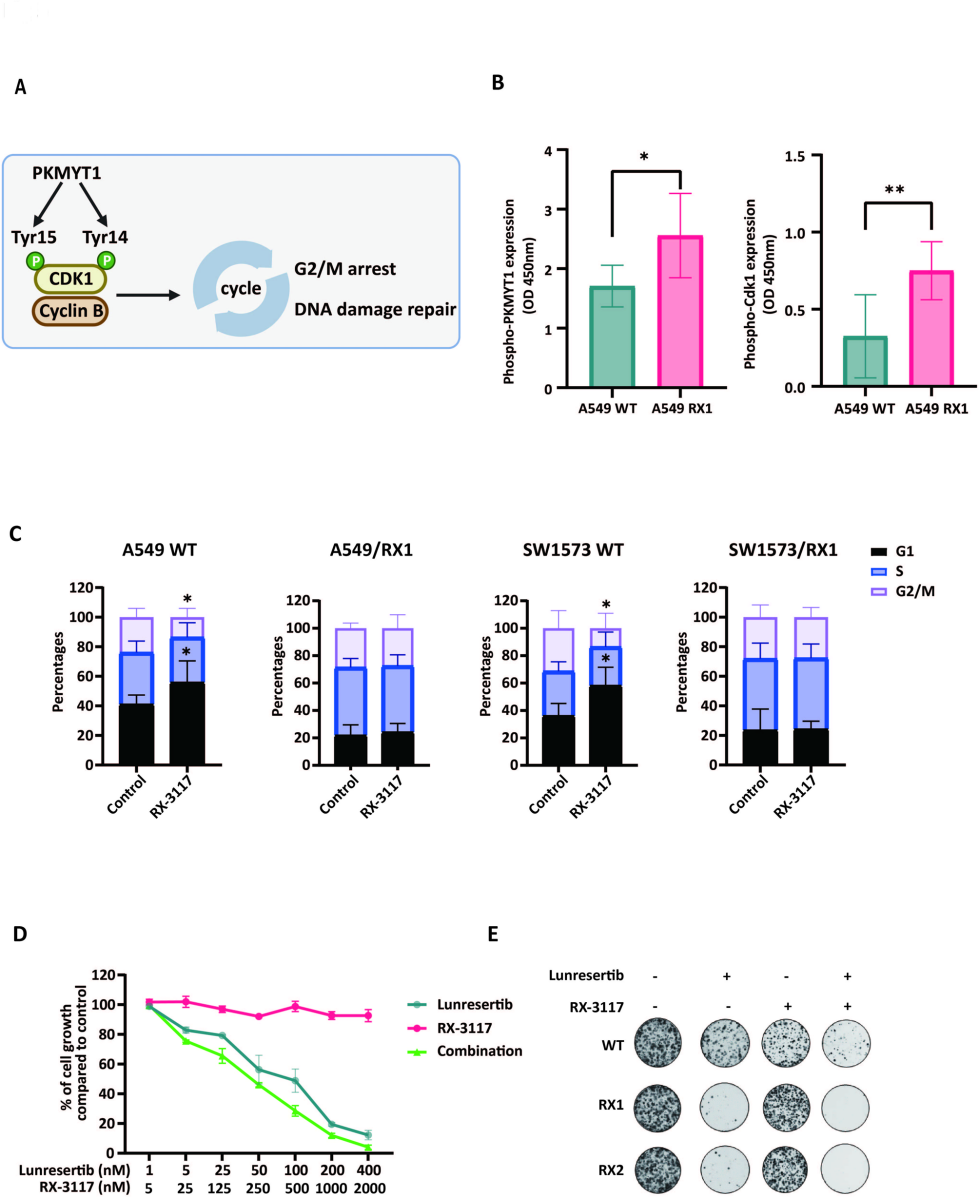
Differential perturbation of cell cycle induced by RX-3117 in RX-3117 resistant cells and inhibitory effects of lunresertib. (**A**) Graphical representation of the role of PKMYT1 on the regulation of cell cycle. (**B**) Enzyme-Linked Immunosorbent Assay (ELISA) of phospho-PKMYT1 and phospho-CDK1 in A549 WT and resistant model RX1. (**C**) Effect of RX3117 on the cell cycle distribution of A549 WT; A549 RX1; SW1573 WT; and SW1573 RX1 cells. (**D**) Growth inhibition curves of A549-RX1 resistant cells following exposure to lunresertib alone, RX3117 alone, or their combination. (**E**) Colony formation assay performed on A549-WT and RX1 cells treated with the 4X IC50 concentration of lunresertib and RX3117. Statistical significance was highlighted by asterisks, as follows: *P < 0.05; **P < 0.01

PKMYT1 plays a role in the cell cycle regulation since it belongs to the WEE1 protein kinase family. PKMYT1 negatively regulates CDK1 by phosphorylating it on two residues (Threonine 14 and Tyrosine 15), leading to the inactivation of the CDK/Cyclin complex and thereby blocking the cell cycle [Fig. 5A] [41]. To further investigate, we examined the expression levels of phosphorylated PKMYT1 and phosphorylated CDK1 using specific ELISA assays. The results showed that phosphorylated PKMYT1 was significantly elevated in the resistant variant of A549 cells, and the expression of phosphorylated CDK1 also increased [Fig. 5B]. Similar results were observed compared in the resistant variants of SW1573, and SW1573/G- cells [Fig. S4].

According to our previous data, we observed clear effects of RX-3117 on cell cycle perturbation in wild type NSCLC cells [42], while, these effects were lacking in resistant variants, supporting an evident role for cell cycle genes in the response to RX-3117 [Fig. 3C]. In particular, wild-type cells were treated with 1 µM RX-3117 for 24 h, while the resistant models were treated with 10 µM RX-3117, with untreated cells serving as controls. In both wild-type A549 and SW1573 cells, RX-3117 treatment resulted in an increase in the G1 phase and a significant reduction in the G2/M phase [Fig. 5C]. However, these changes were not observed in the resistant models [Fig. 5C].

Overall, these findings highlight a distinct effect of RX-3117 on cell cycle distribution in wild-type A549 and SW1573 cells compared to the RX-3117-resistant variants, and are in agreement with the transcriptomics results showing substantial changes in the expression of cell cycle genes in the resistant cells [Fig. 3D].

### Pharmacological validation of the role of PKMYT1 in RX-3117 sensitivity: Inhibition of cell growth and colony formation in A549 resistant cells by lunresertib

For further validation of the role of PKMYT1, using pharmacological tools, we studied the effect of lunresertib, a small molecule inhibitor of PKMYT1 [43], on both A549 parental and resistant cells RX1. To evaluate the IC_50_ of lunresertib, we treated A549 cells with various concentrations of this PKMYT1 inhibitor [Fig. 5D]. As shown in Fig. 5D, resistant cells were sensitive to the drug, with the IC50 for wild-type A549 cells being 206.4 nM, while for RX1 it was 70.68 nM [Fig. S5]. Importantly, the combination of lunresertib with RX-3117 further reduced cell proliferation, and the CI analysis showed a strong synergistic interaction in the resistant cells [Fig. S5], further supporting the key role of PKMYT1 in RX-3117 sensitivity and in overcoming RX-3117 resistance.

Subsequently, we performed a colony formation assay to assess whether the resistant cells were sensitive to the inhibitor. As shown in Fig. 5E, both resistant models exhibited sensitivity to lunresertib, and its combination with RX-3117 was particularly effective in reducing colony formation.

### PKMYT1 expression correlates with poor patient survival in lung cancer

**Fig. 6:**
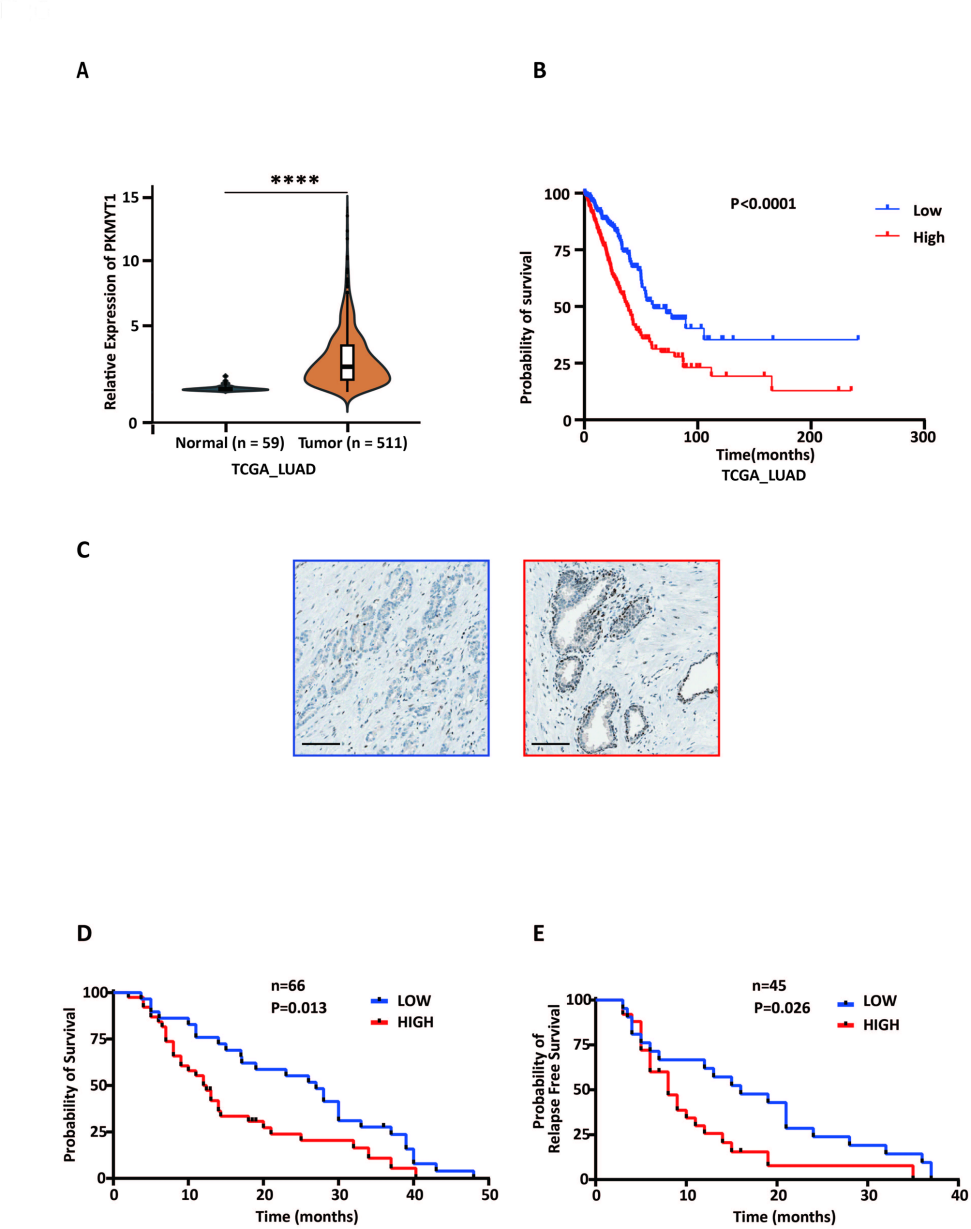
PKMYT1 expression and survival analysis in lung cancer patients. (**A**) Graphs reporting the mRNA expression levels of PKMYT1 in lung cancer tissues and normal tissues in TCGA-LUAD. (**B**) Kaplan–Meier analysis of overall survival in the patients of the TCGA-LUAD cohort (*n* = 502), stratified according to the high or low PKMYT1 mRNA expression levels. (**C**) Representative pictures of PKMYT1 protein staining in NSCLC tissues (left panel showing low expression, right panel showing high expression. Scale bars: 100 μm). (**D**) Kaplan–Meier analysis of overall survival in NSCLC patients (*n* = 66) stratified according to the high or low PKMYT1 protein expression levels. (**E**) Kaplan–Meier analysis of relapse-free survival in NSCLC patients (data on relapse were available for *n* = 45 patients, treated with gemcitabine-based regimens) stratified according to the high or low PKMYT1 protein expression levels

In the TCGA databases, PKMYT1 expression was significantly higher in both LUAD [Fig. 6A] and LUSC [Fig.S6A] lung cancer tissues compared to normal lung tissues. Subsequent Kaplan-Meier analysis revealed that PKMYT1 expression was negatively correlated with overall survival in the TCGA-LUAD cohort [Fig. 6B], but not in TCGA-LUSC cohort [Fig. S6B].

We further investigate PKMYT1 expression at protein level, and Fig. 6C illustrates the differential expression of PKMYT1 in IHC staining images of NSCLC tissues. Table 2 provides the clinicopathological characteristics of our internal patient cohort, which helped to correlate molecular findings with clinical outcomes, such as the impact of PKMYT1 expression on survival. Remarkably, our studies on clinical specimens and data demonstrated that PKMYT1 expression was negatively associated with both overall survival (*P* = 0.01) and relapse-free survival (*P* = 0.03) in NSCLC patients [Fig. 6D and E]. Interestingly, although the sample size was limited, PKMYT1 protein expression correlated with survival in both LUAD and LUSC cases, as reported in the Supplemental Fig.S6C and S6D.

**Table 2:** Clinicopathological characteristics. Main clinical and pathological features of the NSCLC patients whose specimens were stained to investigate PKMYT1 expression levels

Clinicopathological characteristics	*N* (66)	%
**Age**, median (mean ± SD), years	63.5 (62.3 ± 8.6)	
**Sex**		
Male	40	61.6
Female	26	39.3
**Histology**		
ADC	43	65.1
SCC	23	34.8
**Stage**		
IA-IB	8	12.1
IIA	10	15.1
IIB	29	44.9
IIIA	19	29.7
**PS**		
0	27	40.9
1	34	51.5
2	5	7.5
**Smoking history**		
Yes	36	73.4
No	13	26.5

Abbreviations: ADC, Adenocarcinoma; PS, performance status; SCC, Squamous cell carcinoma; SD, Standard deviation

These findings suggest that PKMYT1 may serve as both a prognostic marker and a potential therapeutic target for NSCLC patients.

## Discussion

Nucleoside analogues remain pivotal in the treatment of various cancers, including NSCLC. However, the emergence of drug resistance poses a significant challenge to improving patient survival. Despite their importance, the mechanisms driving drug resistance to these therapies remain poorly understood [44, 45].

In this study, we established, for the first time, multiple NSCLC cell line models with acquired resistance to the new nucleoside analog RX-3117, providing a valuable tool for understanding resistance mechanisms and exploring new potential therapeutic strategies.

The finding that RX-3117-related activating or inactivating enzymes are not associated with resistance is of interest, as it challenges assumptions about traditional resistance mechanisms for nucleoside analogs [1]. Conversely, RX-3117-resistant cells exhibited abnormal expression of cell cycle and DNA repair functions. Moreover, the identification of PKMYT1 activation as a potential driver of resistance is compelling and opens new avenues for targeted research. Targeting PKMYT1 with the first-in-class selective PKMYT1 inhibitor, lunresertib, which entered clinical trials in 2021 [46], was indeed particularly effective in cells resistant to RX-3117. Lastly, analysis of TCGA-LUAD data as well as of an internal cohort of NSCLC patients revealed a correlation between PKMYT1 mRNA and protein expression and poor prognosis. These findings highlight PKMYT1 as a potential prognostic marker and therapeutic target, suggesting that its inhibition could serve as a strategy to fight subpopulations of aggressive and chemoresistant cells in NSCLC.

Understanding the potential mechanisms of nucleoside analog resistance is critical as it can adjust treatment strategies to provide better clinical care [47]. Previous studies on nucleoside analogue resistance have revealed that reduced accumulation of nucleoside analogs and abnormal expression of key enzymes are common mechanisms of cellular resistance [47, 48]. For example, reduced nucleoside transport and decreased activating enzyme UCK2 are major causes of resistance to ETC/TAS-106, Aza-C, and CPEC [49–52]. Inactivation and DNA repair enzymes (NUDT1, DCTPP1, and NT5C3) are upregulated in cytidine analog-resistant cells, which are substrates for these enzymes as well [33, 36, 53, 54]. We observed a significant reduction in the accumulation of nucleotides in resistant cells. Thus, we speculated that this may be due to reduced nucleoside activating enzymes or increased inactivation enzymes, but our results showed no significant or consistent changes in the activating enzymes UCK2, CMPK1, NME1, RR1, and RR2 across our resistant cell models.

This finding contrasts with previous studies, including our own, which have implicated RR in resistance to gemcitabine [55]. These discrepancies may be attributed to differences in the drugs being studied, as well as variations in cellular models and methods used to establish drug resistance. Importantly, the consistency of our current results across at least two independently generated resistant models, developed with a long interval, underscores the robustness and reproducibility of our findings.

Among the inactivating enzymes, DCTPP1 and MTH1 showed no changes, and NT5C3 was the only inactivation enzyme with increased expression in resistant cells. In a pharmacogenetic study, overexpression of this catabolic enzyme of gemcitabine emerged a significant negative predictive factor in NSCLC patients treated with gemcitabine-based therapy, while in vitro studies showed that its silencing or pharmacological inhibition increased the cytotoxicity of gemcitabine [36]. However, in our resistant models blocking NT5C3 does not enhance the inhibitory ability of RX-3117. Similarly, siNT5C3 induced a statistically significant but biologically negligible reduction (less than 15%) in cell proliferation. Thus, enzymes related to RX-3117 activation or inactivation may play only a minor role in mediating RX-3117 resistance.

Conversely, we found that DNA repair genes TDP1 and SAMHD1 were highly expressed in RX-3117-resistant cells, suggesting that DNA repair-related genes or pathways may play a major role in the mechanisms underlying resistance to RX-3117. To further explore if RX-3117 resistance was related with DNA repair or other mechanisms, we performed a genome-wide CRISPR screen and identified PKMYT1 as the gene whose loss increased mostly the sensitivity to RX-3117.

Because the SW1573-resistant variants exhibited a lower fold resistance to RX-3117 compared to the A549 models, we initially focused our omics and mechanistic analyses on the more resistant A549 cells. Nonetheless, despite their relatively lower resistance, particularly in the SW1573/G-/RX variant, the SW1573 models still recapitulate the pivotal role of PKMYT1. Notably, we observed elevated levels of both PKMYT1 phosphorylation and CDK1 phosphorylation across all RX-3117-resistant models. PKMYT1 regulates the cell cycle and DNA repair by phosphorylating the Thr14 and Tyr15 residues of CDK1, inhibiting its activity and preventing premature entry into mitosis [41, 56]. Keeping with this, we observed that RX-3117 sensitizing genes were highly related with DNA repair pathways and cell cycle pathways. For instance, CAND1, UBA3, and CUL3, in addition to PKMYT1, were other three main RX-3117 sensitizing genes, and previous studies linked them to the cell cycle [57–59]. Moreover, we observed that the G2/M checkpoint pathway was activated in the resistant cell lines. The G2/M checkpoint is an important mechanism regulating cell cycle progression, ensuring accurate DNA replication and repairing any DNA damage or replication errors before entering mitosis [60]. GeneMANIA analysis confirmed that PKMYT1 was a hub gene for key genes in the cell cycle pathway. Thus, we speculated that PKMYT1 may lead to RX-3117 resistance by cell cycle disruption and better DNA repair capabilities. The latter was confirmed by our XL-PCR assay.

Previous studies have demonstrated that the inhibition of CDK1 through phosphorylation serves as a critical regulatory mechanism for both the unperturbed cell cycle and the DNA damage checkpoint [61]. Interestingly, while PKMYT1 depletion alone did not significantly impact long-term cell growth, its depletion markedly enhanced the effects of DNA damage, as evidenced by reduced cell growth in clonogenic survival assays and tumor xenograft models. These findings underscore the role of PKMYT1 in checkpoint recovery and position it as a promising target for anti-cancer therapies, particularly in contexts where DNA damage responses are exploited [62]. Other studies showed that the loss of PKMYT1 significantly affected the mitotic index of glioblastoma and human neural progenitor cells [63]. In the present study, PKMYT1 inhibition with lunresertib demonstrated greater efficacy in RX-3117-resistant cells. Moreover, silencing PKMYT1 significantly enhanced the proliferation-inhibitory effects of RX-3117, and the combination of RX-3117 with lunresertib was synergistic. The concurrent modulation of PKMYT1 and CDK1 expression levels align with our hypothesis that PKMYT1 knockout or lunresertib treatment mitigates resistance mechanisms by disrupting enhanced cell cycle regulation and DNA repair processes in resistant cells.

PKMYT1 expression was positively correlated with poor prognosis in various human cancers, such as esophageal squamous cell carcinoma and breast cancer [64, 65]. Consistent with those findings, elevated PKMYT1 expression levels have been observed in lungtumor specimens; however, a significant association with worse survival in the TCGA dataset was confined to the LUAD cohort and not detected in LUSC. The absence of such association in TCGA-LUSC likely reflects the use of RNA data, whereas our own cohort, assessed at the protein level, showed that high PKMYT1 expression was correlated with both shortened overall survival and relapse-free survival in both histological subtypes. This RNA–protein discrepancy parallels the extensive mRNA–protein discordance reported by Satpathy et al. [66], where several cellcycle and DNArepair factors (although PKMYT1 was not specifically assessed) were prognostic only at the protein level. Importantly, our analysis also included a subset of patients treated with gemcitabinebased regimens for whom relapse-free survival data were available; in this group, high PKMYT1 expression was associated with significantly shorter relapse-free survival, suggesting a potential predictive role in treatmentrelevant settings. However, prospective, randomized trials are required to confirm the prognostic and predictive value of PKMYT1 in NSCLC.

To our knowledge, this is the first study to reveal the association between PKMYT1 activation and resistance to a nucleoside analog and to identify that resistant cells are especially sensitive to drug targeting this protein, suggesting that PKMYT1 might be a promising target for lung cancer treatment. A limitation of this study is that we did not evaluate whether PKMYT1 could be a common resistance marker for other nucleoside analogs.

Currently, research on PKMYT1 inhibitors is limited, with lunresertib being the only reported effective PKMYT1 inhibitor, capable of inducing synthetic lethality in cancers with CCND1 amplification [43, 65]. This drug showed relatively low IC50 values in our NSCLC cells. Preliminary data of the Phase I MINOTAUR trial of this drug in combination with FOLFIRI in advanced gastrointestinal cancers showed a safety profile consistent with FOLFIRI alone, and promising efficacy, suggesting that this drug could provide a safe and effective therapeutic approach [67].

However, further studies including both in vitro and in vivo models of NSCLC are needed. Of note, the models we used are carrying *K-Ras* mutations (G12S, and G12C in A549 and SW1573 cells, respectively) [68]. Considering the emergence of new anti-RAS targeted therapies [69], future studies should use models which do not harbor such mutations or test potential combinations of K-Ras and PKMYT1 inhibitors.

## Conclusion

In summary, by integrating CRISPR-Cas9 technology with transcriptomics, functional assays, and pharmacological evaluations across multiple cellular models of resistance, we established a robust framework for unraveling resistance mechanisms to the novel nucleoside analog RX-3117. With such framework, we demonstrated that aberrant DNA repair and cell cycle dysregulation drive such resistance, identifying PKMYT1 as a key contributor. These findings position PKMYT1 as a promising therapeutic target and warrant further studies, particularly in patients with high PKMYT1 expression, which is significantly associated with reduced survival rates in NSCLC.

## Electronic supplementary material

Below is the link to the electronic supplementary material.

Supplementary Material 1

Supplementary Material 2

Supplementary Material 3

Supplementary Material 4

Supplementary Material 5

Supplementary Material 6

Supplementary Material 7

Supplementary Material 8

Supplementary Material 9

## Data Availability

Sequencing data that support the findings of this study are publicly available in the Gene Expression Omnibus (GEO) database under the accession number GSE300920. The cells which are not available through public repositories can be requested from the corresponding authors, subject to a Material Transfer Agreement. Data analyzed during this study are included in the main text or in the Supplementary section. Raw data are stored in the laboratory of the corresponding authors and are available upon request.
