# Supplementary material for: Unraveling resistance mechanisms to the novel nucleoside analog RX-3117 in lung cancer: insights into DNA repair, cell cycle dysregulation and targeting PKMYT1 for improved therapy
Source: J Exp Clin Cancer Res. 2025 Jul 24;44:217. doi: 10.1186/s13046-025-03470-z (PMC12288264; doi:10.1186/s13046-025-03470-z)
Supplement: Supplementary file 1 — Supplementary Material 1 [file 13046_2025_3470_MOESM1_ESM.docx]

**ADDITIONAL FILES**

**Additional file 1: Supplementary Methods**

**Evaluation of inhibition of cell growth using the sulforhodamine B (SRB) assay**

Cellular sensitivity to RX-3117, gemcitabine, 5-azacytidine (AzaC), and lunresertib (RP-6306), was assessed using the SRB assay. A total of 3,000–5,000 cells per well were seeded into 96-well plates and treated with the compounds for 72 hours. After fixation with 25μL/well cold 50% trichloroacetic acid (TCA, final concentration 5.6%), and SRB staining with 10 mM Tris base solution, the optical density (OD) was measured with a BioTek Synergy HT plate reader (BioTek Instruments Inc., Winooski, USA) at a wavelength of 490 nm. Cell growth inhibition was calculated as the percentage OD of drug-treated cells versus that of vehicle-treated cells (negative control) corrected for OD before drug addition, day-0. The half maximal-inhibitory concentration (IC50) was calculated by non-linear least-squares curve fitting with GraphPad Prism version 9 (Intuitive Software for Science, San Diego, USA), or interpolation when the curve was not sigmoidal.

**Studies on the effects of inhibition of NT5C3 on the inhibition of cell growth by RX-3117**

To investigate whether the gene expression variability of NT5C3 observed between tumor tissues might affect the cytotoxic activity of RX-3117 we evaluated the effects of a specific siRNA (Assay ID#36451, ThermoFisher Scientific, Waltham, MA, USA) against NT5C3 in A549 cells. Cells were plated at 10^5^ cells per well in 6-well plates and, after 24 hours, the cells were transfected with small interfering RNA (siRNA) oligonucleotide or negative-control siRNA using Oligofectamine (Thermofisher #12252011) to result in a final RNA concentration of 5 nmol/l in serum-free medium, according to the manufacturer’s instructions. The expression level of NT5C3 was studied with quantitative PCR. After transfection, the cells were treated with RX-3117 (1 μM) for 24 hours. The cell growth inhibitory effect of RX-3117 was studied by direct cell count using the trypan blue. Growth inhibition was expressed as the percentage of RX-3117-untreated controls (untransfected and negative-control siRNA-treated cells).

Additional pharmacological studies were performed with the NT5C3 inhibitor diethylpyrocarbonate (DEPC). Cells were plated in 96-well plates, and treated with RX-3117 for 72 hours alone or in combination with 10 μM DEPC. The cell growth inhibitory effects were studied using SRB assay.

**Measurement of nucleotide accumulation by LC-MS/MS**

Parental or resistant cells, either not transfected, or transfected with negative-control siRNA (Scramble), or with siRNA targeting NT5C3 (siNT5C3), as described above, were treated with 1 or 10 µM RX-3117 for 24 hours, washed twice with phosphate-buffered saline (PBS), and pelleted by centrifugation at 350 × g at 4°C for 5 minutes. Pellets were snap-frozen in liquid nitrogen and stored at -80°C until analysis. For LC-MS/MS analysis, pellets were resuspended in 200 μL water, with 20 μL used for protein content determination using the modified Pierre BCA method. The remaining 180 μL was precipitated with isopropyl alcohol, centrifuged at 2500 × g at 4°C, and the supernatant freeze-dried and reconstituted in water. A 20 μL aliquot of the reconstituted sample was used to measure free cytosolic RX-3117, while the remaining sample was treated overnight at 37°C with alkaline phosphatase (4 units) to degrade RX-3117 nucleotides to free RX-3117 (19, 20). Following sample cleanup and freeze-drying, total RX-3117 content (degraded nucleotides and free RX-3117) was analyzed using LC-MS/MS. Chromatographic separation was performed on a Prodigy 5 ODS-2 column (150 × 3.2 mm, Phenomenex), and detection was conducted under optimized ElectroSpray Ionization conditions with an API5500 Triple Quadrupole mass analyzer (AB Sciex, Netherlands BV). Measurements were conducted in triplicate, and total phosphorylated RX-3117 levels were determined by subtracting free RX-3117 from the total RX-3117 measured in the second analysis.

**Colony formation assay**

Cells were seeded in 6-well plates 3000 -5000 cells per well to provide adequate space for colony formation. Following a 24-hour adhesion period, cells were treated with RX-3117 and lunresertib, individually or in combination. After treatment, cells were fixed with methanol for 10 minutes at room temperature to preserve colony morphology. Following fixation, colonies were stained with 0.5% crystal violet solution (w/v in water) for 15–20 minutes. Excess stain was removed by gently washing with water, and the plates were left to air dry completely. Colonies consisting of at least 50 cells were counted manually or using image analysis software. The results were expressed as the percentage of colony formation relative to untreated controls.

**Cell cycle distribution**

Cells were seeded in 10-cm dishes and treated for 24 hours with 1 μM RX-3117 for A549, SW1573, and 10 μM for the resistant variants A549/RX1 and SW1573/RX1. After treatment, the medium was collected, and the dishes were washed twice with PBS followed by trypsinization for 3-5 minutes. Detached cells were resuspended in medium, collected, and stored on ice. The samples were then spun down at 2000 rpm for 5 minutes at 4°C with two washing steps in cold PBS. For each sample, 2x10⁵ cells were fixed in 500 µL of ice-cold 70% ethanol while vortexing and incubated for at least 1 hour at 4°C. Ethanol was removed by centrifugation, and the cells were washed with cold PBS before another centrifugation. A total of 100 µL of FxCycleTM propidium iodide (PI)/RNase staining solution was added, and the samples were incubated in the dark at room temperature for at least 30 minutes. After proper intercalation of PI and RNA degradation by RNase, the stained samples were analyzed on an Attune NxT Flow Cytometer (Thermo Fisher Scientific). The flow cytometer, equipped with blue (488 nm) and yellow (561 nm) lasers, was used to detect PI fluorescence. Data acquisition and analysis were conducted using Attune NxT software, with at least 10,000 events per sample collected, and debris or doublets excluded.

**Analysis of PKMYT1 by immunohistochemistry (IHC)**

Formalin-fixed, paraffin-embedded specimens from patients who underwent radical surgical resection for primary NSCLC between 2004 and 2014 were used to create tissue microarrays (TMAs), using a TMA instrument (Beecher Instruments, Micro-Array Technologies). The TMA sections were de-paraffinized with xylene, rehydrated in alcohol, and subjected to antigen retrieval in EDTA using a pressure cooker. Sections were then incubated overnight with a rabbit monoclonal antibody specific for endogenous PKMYT1 (Cell Signaling Technology #4282), followed by incubation with a horseradish peroxidase-conjugated secondary antibody at room temperature for 60 minutes. The antigen-antibody complex was visualized by incubating the sections with 3,3'-diaminobenzidine (DAB), which resulted in a brown precipitate at the staining site. The sections were rinsed, counterstained, and mounted for microscopic evaluation. IHC results were analyzed semi-quantitatively based on staining intensity and the extent of positive staining. DAB staining intensity was graded as 0 for negative, 1 for light yellow, 2 for light brown, and 3 for dark brown. The percentage of positively stained cells was scored from 1 to 4, ranging from 0-25% to 76-100% positive cells. The overall IHC-score was calculated by adding the staining intensity and the proportion of positively stained cells, offering a comprehensive assessment of PKMYT1 expression.

**Additional file 2: Fig. S1. Modulation of RX-3117 nucleosides and antiproliferative activity of RX3117 by NT5C3 silencing in A549 cells.** (A), Densitometric analysis of Western blot bands of metabolism enzymes. Quantification was performed using ImageJ (NIH, Bethesda, MD, USA) by measuring the mean grey value of inverted band images. Background-corrected intensities are expressed as the ratio of target protein to housekeeping protein (i.e. Relative Expression of Protein). (B) Expression level of NT5C3 by quantitative PCR after specific silencing in A549 WT compared to siRNA negative control (Scramble), (C) Modulation of accumulation of RX-3117 nucleotides in A549 WT, and RX-3117 resistant cells after transfected with negative-control siRNA (Scramble), or with siRNA targeting NT5C3 (siNT5C3), (D), Cell growth of cells treated with RX-3117 (1 µM) after exposure to the NT5C3 inhibitor diethylpyrocarbonate (DEPC) and siRNA-NT5C3. Cell growth of treated cells was compared to growth of untreated control cells set at 100%. (E) Densitometric analysis of Western blot bands of DNA repair enzymes, performed as described above. Data represent mean ± SD from at least three independent experiments.

**Additional file 3: Fig. S2. Top enriched gene sets of desensitizing genes in A549 cells treated with RX-3117.** GSEA analysis was used to identify the 15 most enriched gene sets of desensitizing genes in A549 cells treated with RX-3117.

**Additional file 4: Fig. S3. Top 15 pathway enriched in the RNA-seq data between wild type (WT) cells and RX-3117 resistant cells, as determined by the GSEA analysis.** (A) SW1573-WT cells and SW1573-RX1 cells, (B) H460-WT cells and H460-RX cells.

**Additional file 5: Fig. S4 Modulation of phospho-PKMYT1 and phospho-CDK1 in SW1573 models.** (A), Enzyme-Linked Immunosorbent Assay (ELISA) of phospho-PKMYT1 in SW1573-WT and SW1573-RX1 cells (B) ELISA of phospho-PKMYT1 in SW1573/G- and SW1573/G-/RX1 cells, (C), ELISA of phospho-CDK1 in SW1573-WT and SW1573-RX1 cells (D) ELISA of phospho-CDK1 in SW1573/G- and SW1573/G-/RX1 cells.

**Additional file 6: Fig.S5. Activity of the PKMYT1 inhibitor lunresertib.** (A) Inhibition of cell growth in A549 WT and A549 resistant model RX1 after exposure to different concentrations of lunresertib. (B) Synergistic interaction of lunresertib and RX3117 in A549 RX1 cells: the pharmacological interaction was evaluated by calculating the combination index (CI) in cells exposed to a fixed drug ratio (1:5, based on the IC50s in the WT cells, hypothesizing that the combination would restore the activity of RX-3117 in the RX1 cells), using CalcuSyn.

**Additional file 7: Fig.S6. PKMYT1 expression and survival analysis in lung cancer patients.** (A) Comparison of the expression levels of PKMYT1 in lung cancer (TCGA-LUSC cohort) and normal tissues in (B) Kaplan–Meier analysis of overall survival in patients of the TCGA-LUSC cohort (n=491) grouped according to high or low PKMYT1 expression level. (C) Kaplan–Meier analysis of overall survival in patients with LUAD of the internal cohort (n=43) grouped according to high or low PKMYT1 expression level. (D) Kaplan–Meier analysis of overall survival in patients with LUSC of the internal cohort (n=23) grouped according to high or low PKMYT1 expression level.

**Additional file 8: Table S1. Chemical and biological reagents and materials.** List of drugs, chemicals, antibodies and primers used to perform the experiments described in the current article.

**Additional file 9: Table S2.** **Inhibition of cell growth by cytidine analogs.** Evaluation of the inhibition of cells growth in NSCLC cell lines and their RX-3117 resistant variants to RX-3117 upon treatment with RX-3117 and various cytidine analogs. IC50 values in RX-3117-resistant variants were conducted to assess potential cross-resistance.

Abbreviations: Aza-C, azacytidine; IC_50_, Half maximal inhibitory concentration; SEM, standard error of the mean.
