# Supplementary figures and images for: Unraveling resistance mechanisms to the novel nucleoside analog RX-3117 in lung cancer: insights into DNA repair, cell cycle dysregulation and targeting PKMYT1 for improved therapy
Source: J Exp Clin Cancer Res. 2025 Jul 24;44:217. doi: 10.1186/s13046-025-03470-z (PMC12288264; doi:10.1186/s13046-025-03470-z)

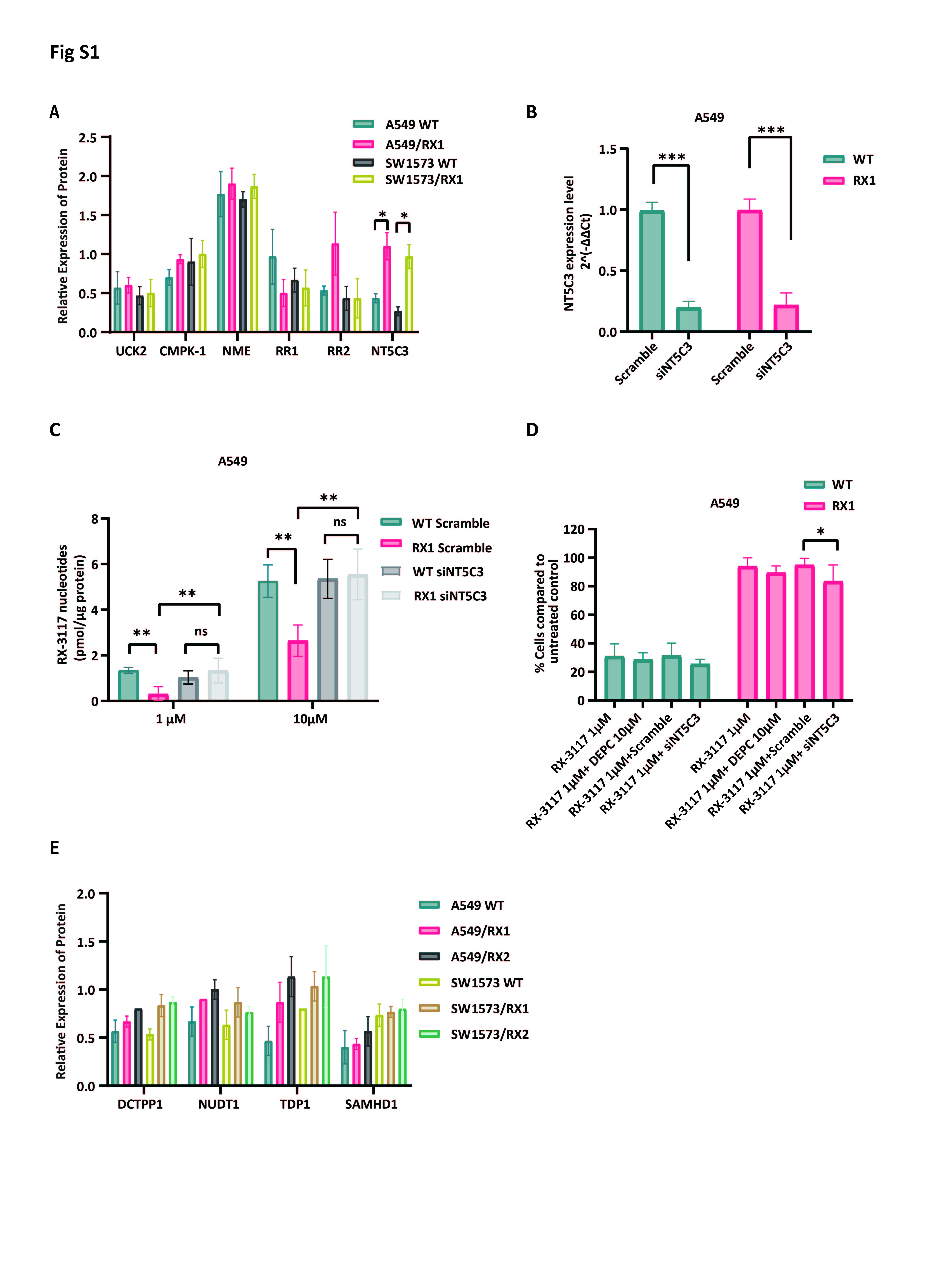

Supplement: Supplementary file 2 — Supplementary Material 2 [file 13046_2025_3470_MOESM2_ESM.jpg]

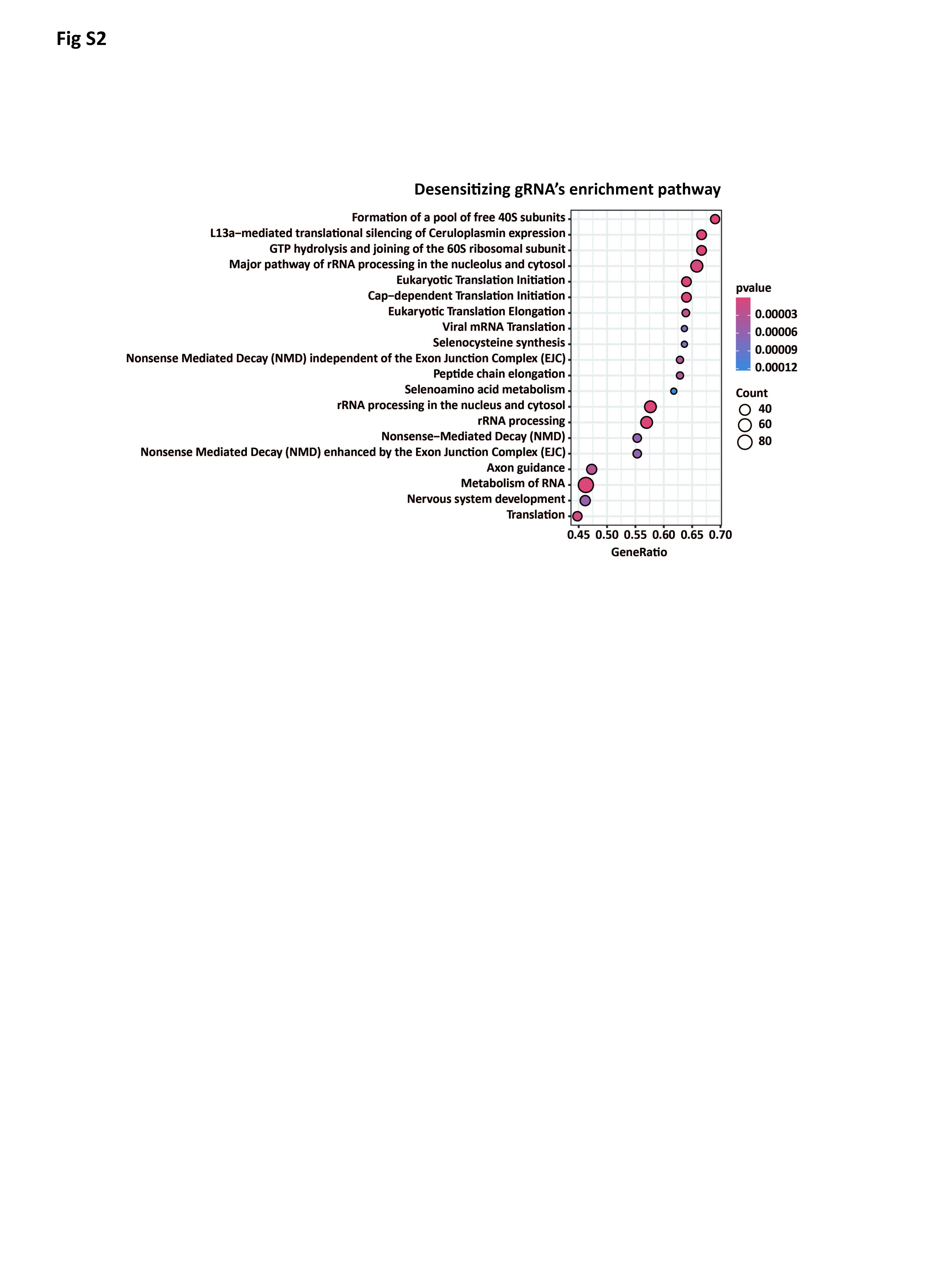

Supplement: Supplementary file 3 — Supplementary Material 3 [file 13046_2025_3470_MOESM3_ESM.jpg]

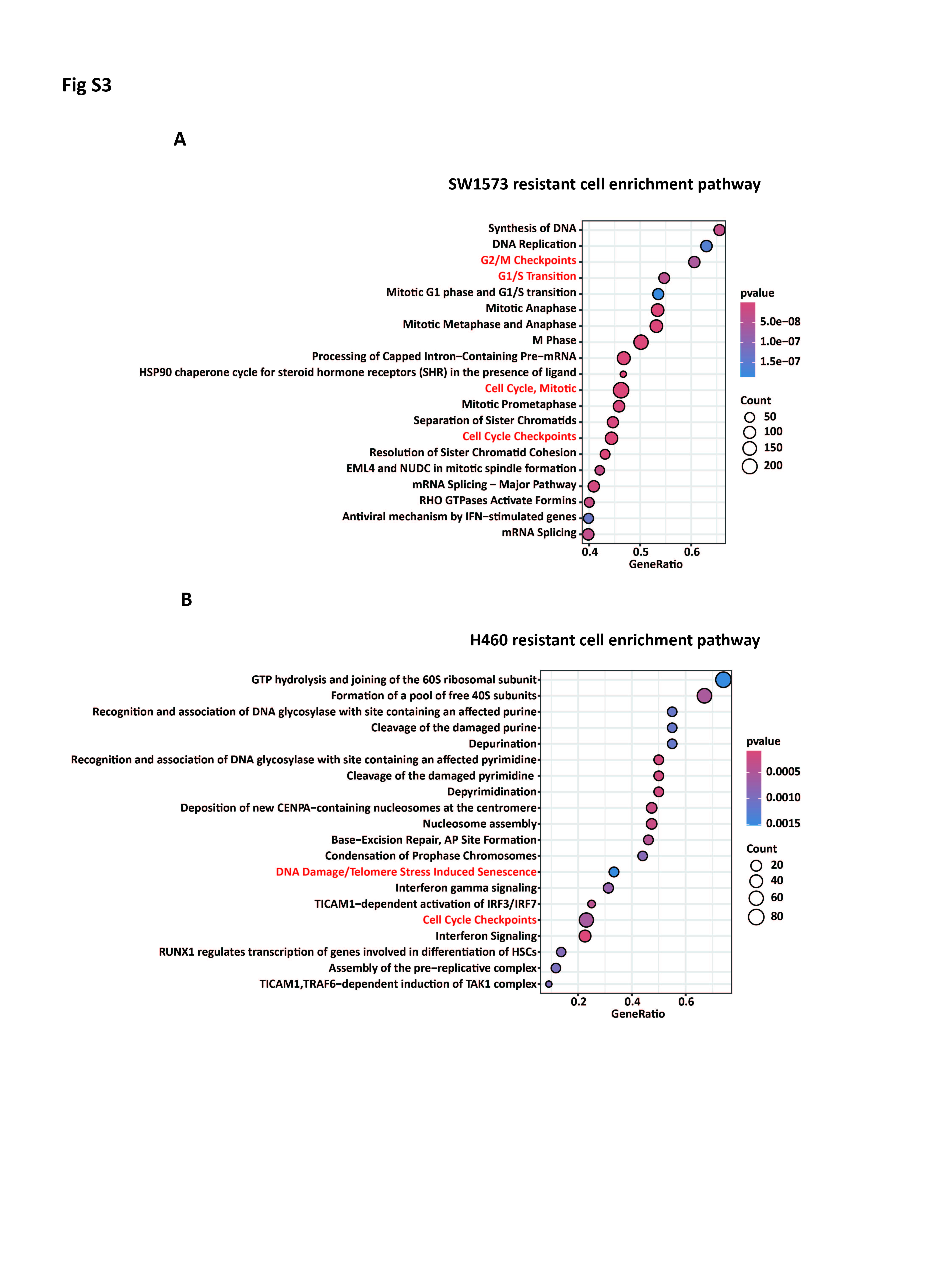

Supplement: Supplementary file 4 — Supplementary Material 4 [file 13046_2025_3470_MOESM4_ESM.jpg]

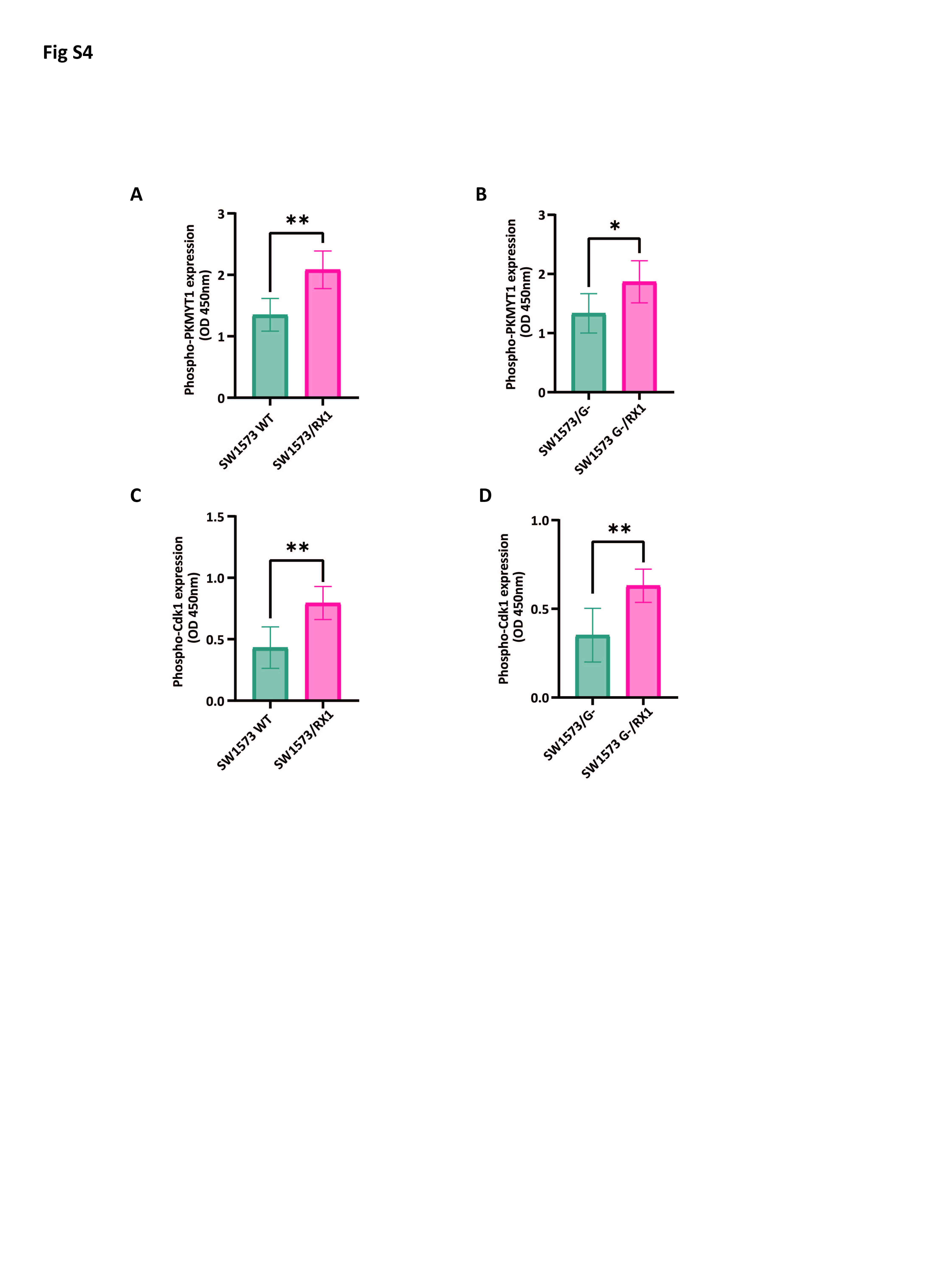

Supplement: Supplementary file 5 — Supplementary Material 5 [file 13046_2025_3470_MOESM5_ESM.jpg]

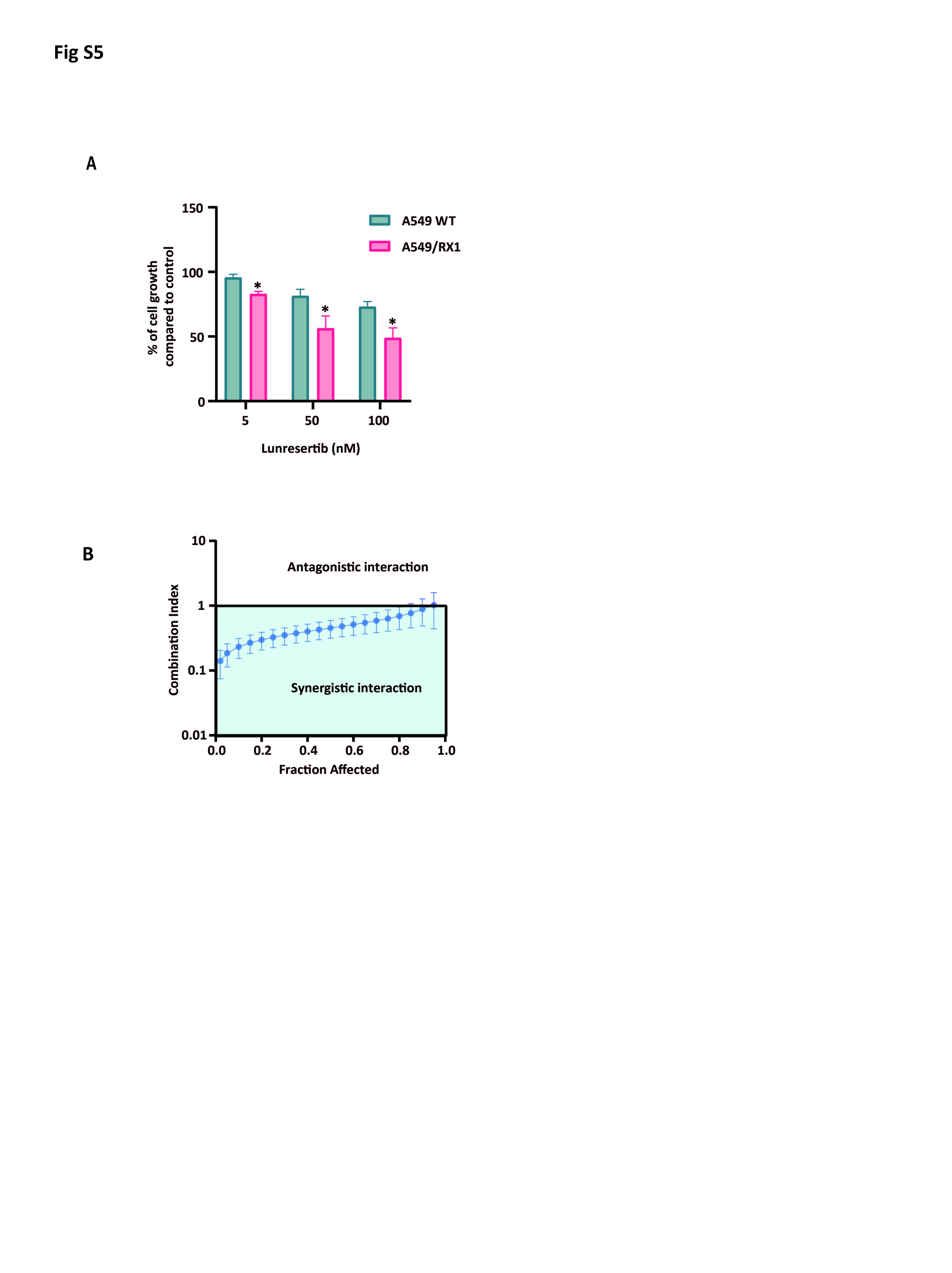

Supplement: Supplementary file 6 — Supplementary Material 6 [file 13046_2025_3470_MOESM6_ESM.jpg]

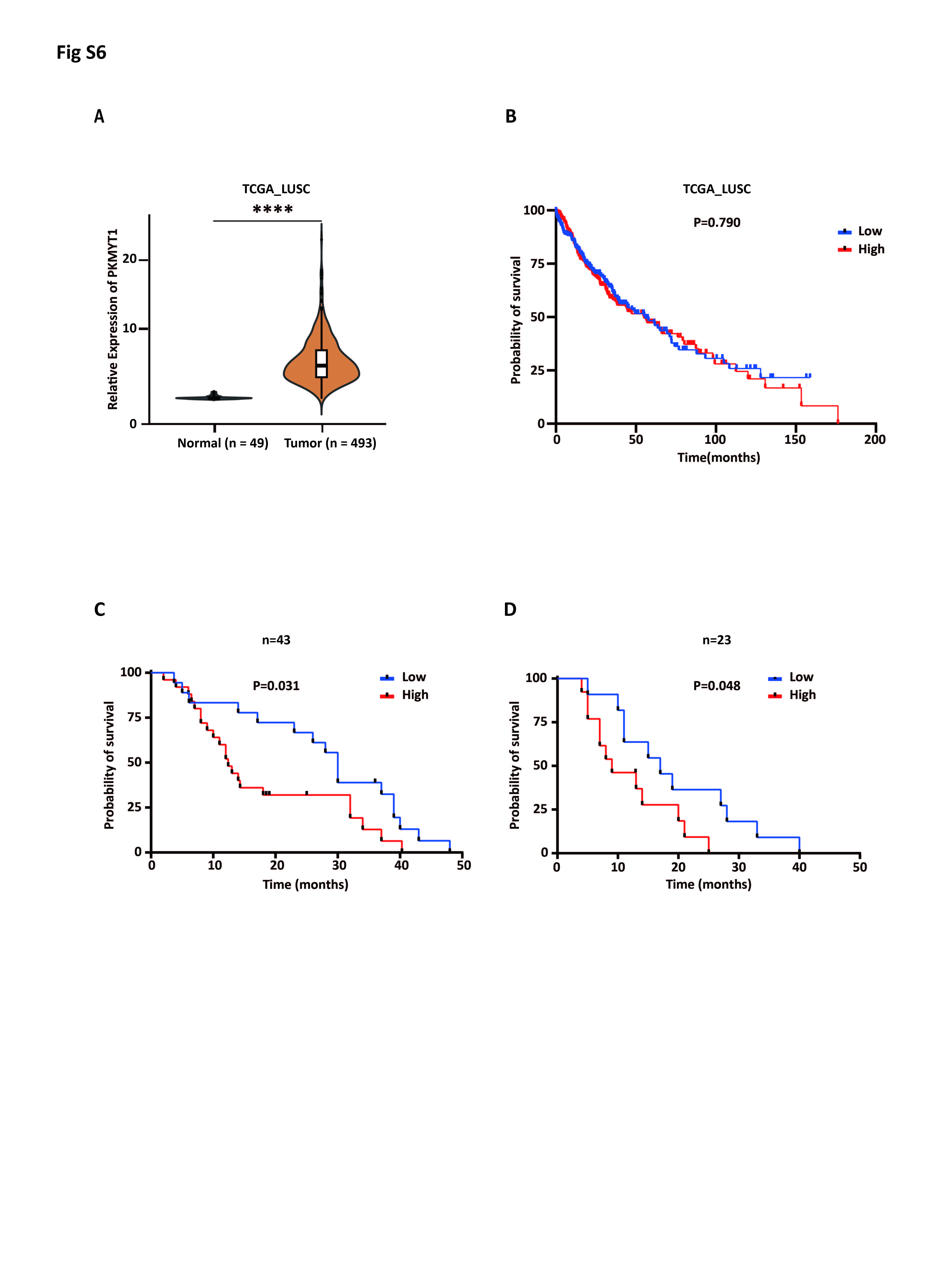

Supplement: Supplementary file 7 — Supplementary Material 7 [file 13046_2025_3470_MOESM7_ESM.jpg]
