# Supplementary material for: Unraveling resistance mechanisms to the novel nucleoside analog RX-3117 in lung cancer: insights into DNA repair, cell cycle dysregulation and targeting PKMYT1 for improved therapy
Source: J Exp Clin Cancer Res. 2025 Jul 24;44:217. doi: 10.1186/s13046-025-03470-z (PMC12288264; doi:10.1186/s13046-025-03470-z)
Supplement: Supplementary file 8 — Supplementary Material 8 [file 13046_2025_3470_MOESM8_ESM.docx]

**Table S1. Chemical and biological reagents and materials.** List of drugs, chemicals, antibodies and primers used to perform the experiments described in the current article.

| **Drugs, chemicals and kits** |  |
| --- | --- |
| DMEM | Gibco, cat# 41965062 |
| FBS | Biowest, S1810-100 |
| Hepes buffer | Lonza, 17-737E |
| Sodium pyruvate | Gibco, cat# 11360070 |
| MycoAlert Mycoplasma detection kit | Westburg, LO LT07-318 |
| RX-3117 | Rexahn pharmaceuticals |
| lysis buffer | Cell signaling, #9803 |
| PMSF | Sigma, P7626 |
| urea buffer | Sigma, 51456 |
| orthovanadate | Sigma, S6508 |
| tetrasodium pyrophosphate | Sigma, S6422 |
| β-glycerophosphate | Sigma, G5422 |
| BSA | Sigma-Aldrich, 05482 |
| Bio-Rad protein assay dye | Bio-Rad, #5000006 |
| MiRVAna | Ambion, Thermo Fisher Scientific |
| Illumina TruSeq Stranded Total RNA Library Prep Gold Kit | Illumina Inc., San Diego, USA, 20020598 |
| Agencount AMPure XP beads | Beckman Coulter, Brea, USA |
| TRIzol | ThermoFisher Scientific,15596-026 |
| First Strand cDNA Synthesis Kit | Thermo Scientific, #K1612 |
| SsoAdvanced Universal SYBR Green Supermix | Bio-Rad, #1725271 |
| Phospho-PKMYT1 (T495) and Total PKMYT1 ELISA Kit | ab279891 |
| Human Phospho-CDK1 (Y15) and Total CDK1 ELISA Kit | Raybiotech,Catalog #: PEL-CDK1-Y15-T-1 |
| FxCycle^TM^ PI/RNase | ThermoFisher scientific, F10797 |
|  |  |
| **Antibodies** |  |
| UCK2 | 1: 1000, monoclonal antibody (mab) 22-1, gift from Rexahn |
| CMPK1 | 1:1000, Origene TAS505390 |
| NME1/NDKA (D14H1) | 1:1000, Cell signaling lot#5353 |
| Ribonucleotide reductase R1 T-16 | 1:132, Santa Cruz sc-11733 |
| Ribonucleotide reductase R2 E-16 | 1:132, Santa Cruz sc-10846 |
| DCTPP1 | 1:1000, LSBio LS-C80879-100 |
| NUDT1/MTH1 | 1:1000, Cell signaling technology lot#43918 |
| TDP1 (D8D1B) | 1:1000, Cell signaling lot#59710S |
| NT5C3 | 1:500, Abcam ab224766 |
| SAMHD1 | 1:1000, Cell signaling lot#12361S |
| β-actin clone AC-15 | 1:2500, Sigma Aldrich A1978 |
| PKMYT1-Myt1 | 1:1000, Cell signaling #4282 |
|  |  |
| **Primer** |  |
| PKMYT1-Forward Sequence | TATGGGACAGCAGCGGATGTGT |
| PKMYT1-Reverse Sequence | AGAACGCAGCTCGGAAGACAGA |
|  |  |
| **gRNA** |  |
| PKMYT1-exon 4 | CAAGGACCGGGCCCGCAAGT |
|  |  |
| **si-RNA** |  |
| siRNA- NT5C3 | Thermo Fsher scientific- Assay ID: 135052  Thermo Fsher scientific- TaqMan Gene Expression Assay, Assay ID: Hs05574347_g1 |
| siRNA-negative control (Scramble) | Thermo Fsher scientific- Catalog Number: AM4611 |
