# Supplementary material for: Unraveling resistance mechanisms to the novel nucleoside analog RX-3117 in lung cancer: insights into DNA repair, cell cycle dysregulation and targeting PKMYT1 for improved therapy
Source: J Exp Clin Cancer Res. 2025 Jul 24;44:217. doi: 10.1186/s13046-025-03470-z (PMC12288264; doi:10.1186/s13046-025-03470-z)
Supplement: Supplementary file 9 — Supplementary Material 9 [file 13046_2025_3470_MOESM9_ESM.docx]

**Table S2.** **Inhibition of cell growth by cytidine analogs.** Evaluation of the inhibition of cells growth in NSCLC cell lines and their RX-3117 resistant variants to RX-3117 upon treatment with RX-3117 and various cytidine analogs. IC50 values in RX-3117-resistant variants were compared conducted to assess potential cross-resistance.

| Cell line |  | RX-3117 (μM) | Gemcitabine (nM) | Aza-C (μM) |
| --- | --- | --- | --- | --- |
| A549 |  | 0.5 ± 0.008 | 6.4 ± 0.4 | 4.2 ± 0.6 |
| A549/RX1 |  | > 400 | > 200 | > 100 |
| A549/RX2 |  | 300 ± 19.7 | 23.1 ± 1.9 | 55.2 ± 3.8 |
|  |  |  | |  |
| SW1573 |  | 0.6 ± 0.15 | 8.3 ± 0.3 | 7.1 ± 1.8 |
| SW1573/RX1 |  | > 400 | 32.7 ± 4.9 | 59.9 ± 22.0 |
| SW1573/RX2 |  | 292 ± 10.2 | 20.4 ± 1.1 | > 100 |
|  |  |  | |  |
| SW1573/G- |  | 1.0 ± 0.1 | > 2500 | 6.5 ± 0.8 |
| SW1573/G-/RX |  | 9.1 ± 1.1 | > 2000 | 15.4 ± 2.2 |

Abbreviations: Aza-C, azacytidine; IC_50_, Half maximal inhibitory concentration; SEM, standard error of the mean.
